# Supplementary material for: Molecular mechanisms of mitochondrial Ca2+ exchanger NCLX
Source: Nat Commun. 2026 Jul 10;17:8523. doi: 10.1038/s41467-026-75483-x (PMC13483046; doi:10.1038/s41467-026-75483-x)
Supplement: Supplementary file 1 [file 41467_2026_75483_MOESM1_ESM.pdf]

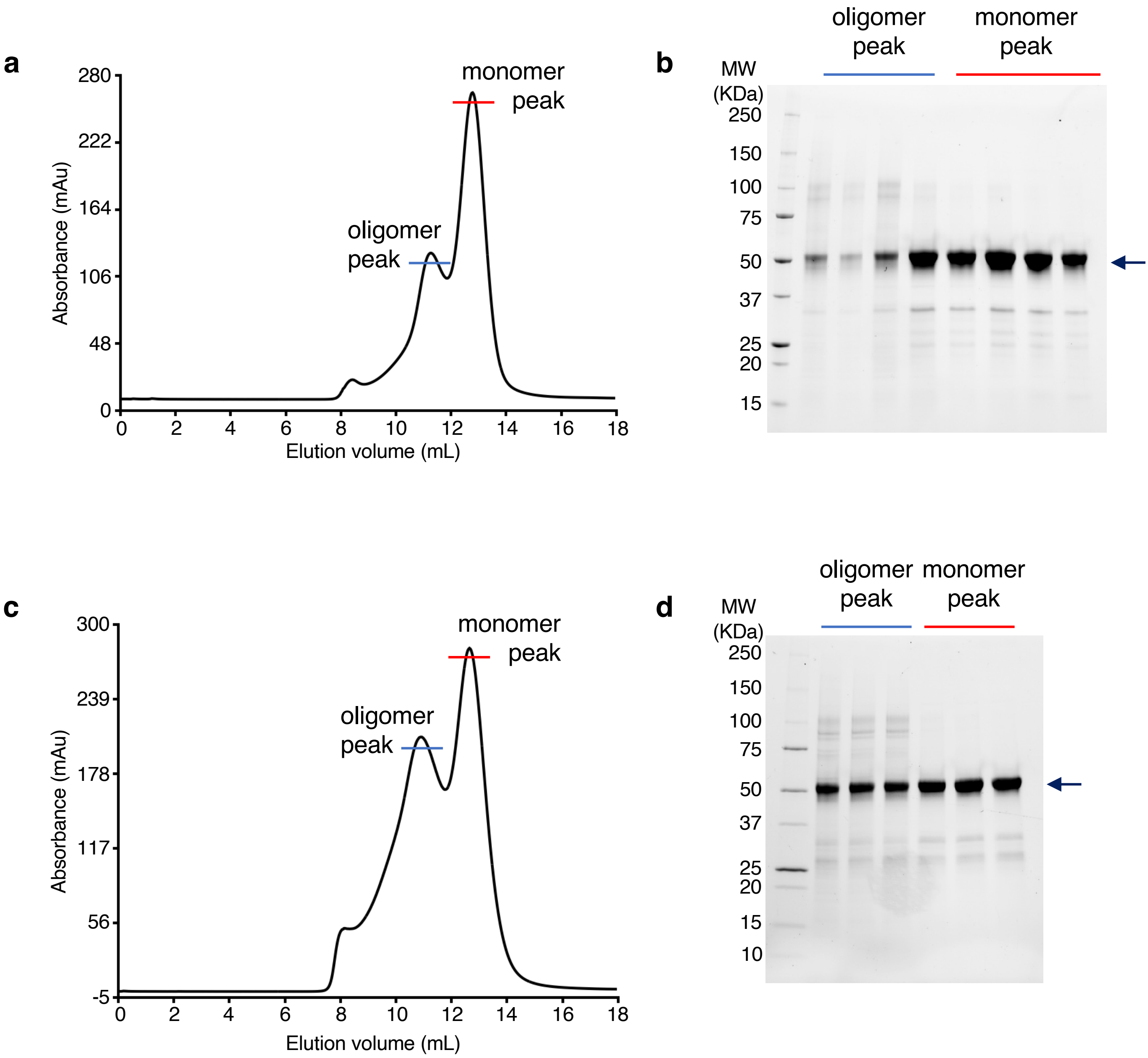

Supplementary Fig. 1 Biochemical analysis of rat NCLX. a Size-exclusion chromatography (SEC) profile of NCLX purified from total cell extracts showing two distinct elution peaks, and **b** SDS-PAGE of fractions collected from the corresponding SEC peaks. The arrow marks the NCLX monomer band in SDS-PAGE. **c** The SEC profile of purified NCLX extracted from the mitochondrial membrane, and **d** SDS-PAGE of fractions collected from the corresponding SEC peaks.

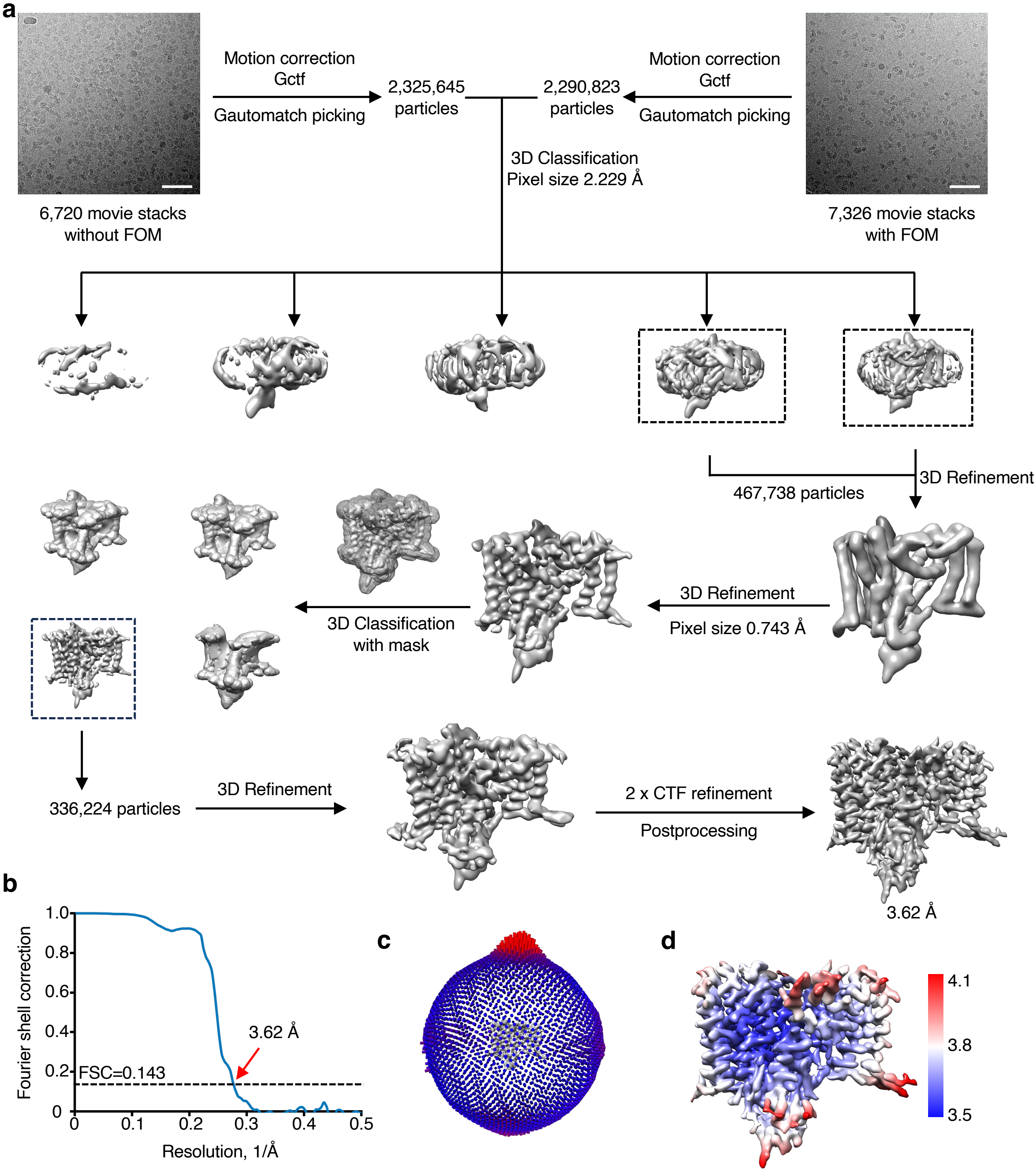

Supplementary Fig. 2 Cryo-EM data processing of monomeric NCLX. **a** Workflow of the cryo-EM data processing. The scale bar in the representative micrograph corresponds to 500 Å. **b** Golden-standard Fourier shell correction (FSC) curve of the 3D reconstruction. **c** Angular distribution of particle projections of the 3D reconstruction. **d** Local resolution of the rat monomeric NCLX estimated using RELION.

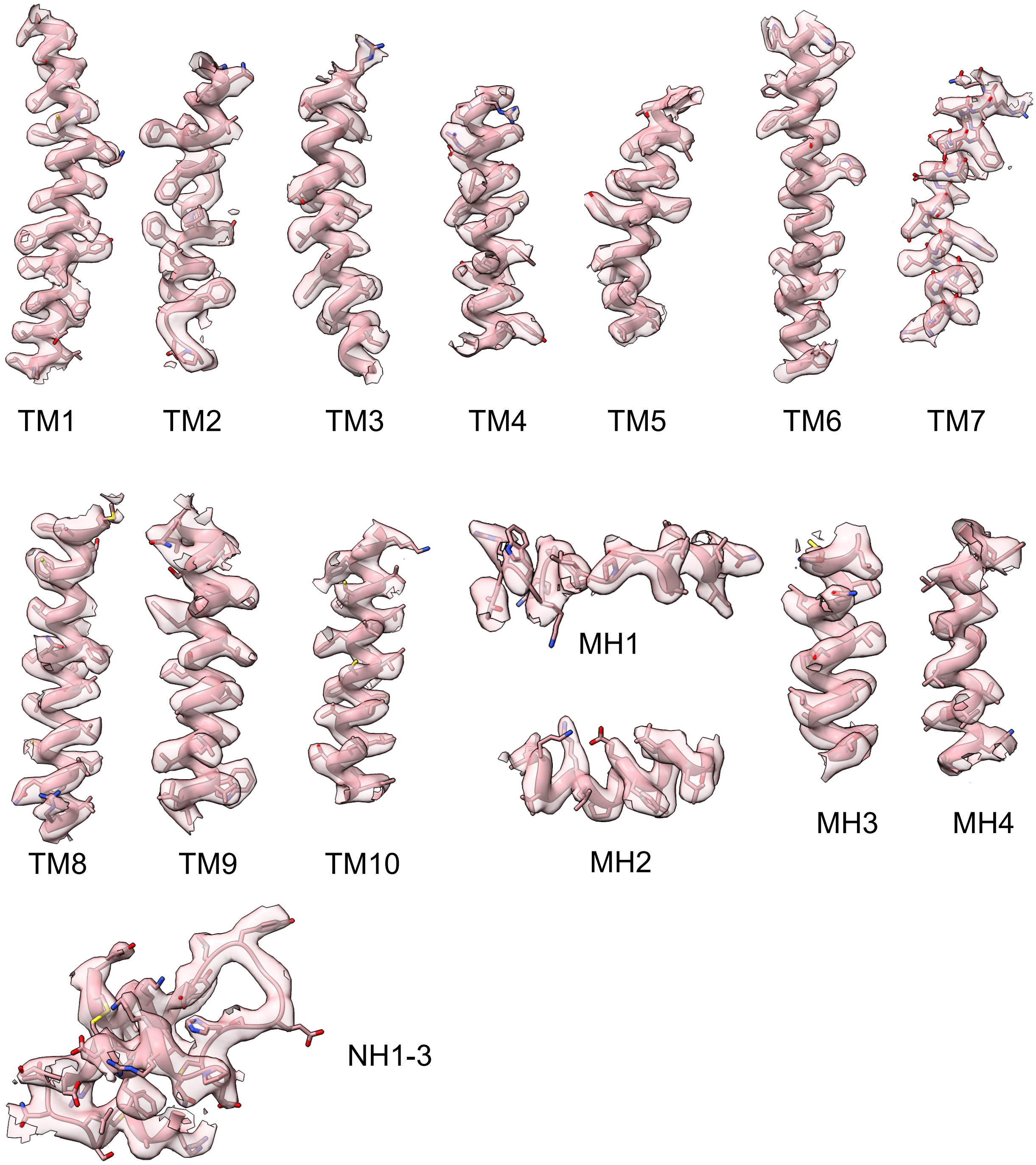

Supplementary Fig. 3 Density map of rat monomeric NCLX. Density maps of rat monomeric NCLX structure contoured at 6σ.

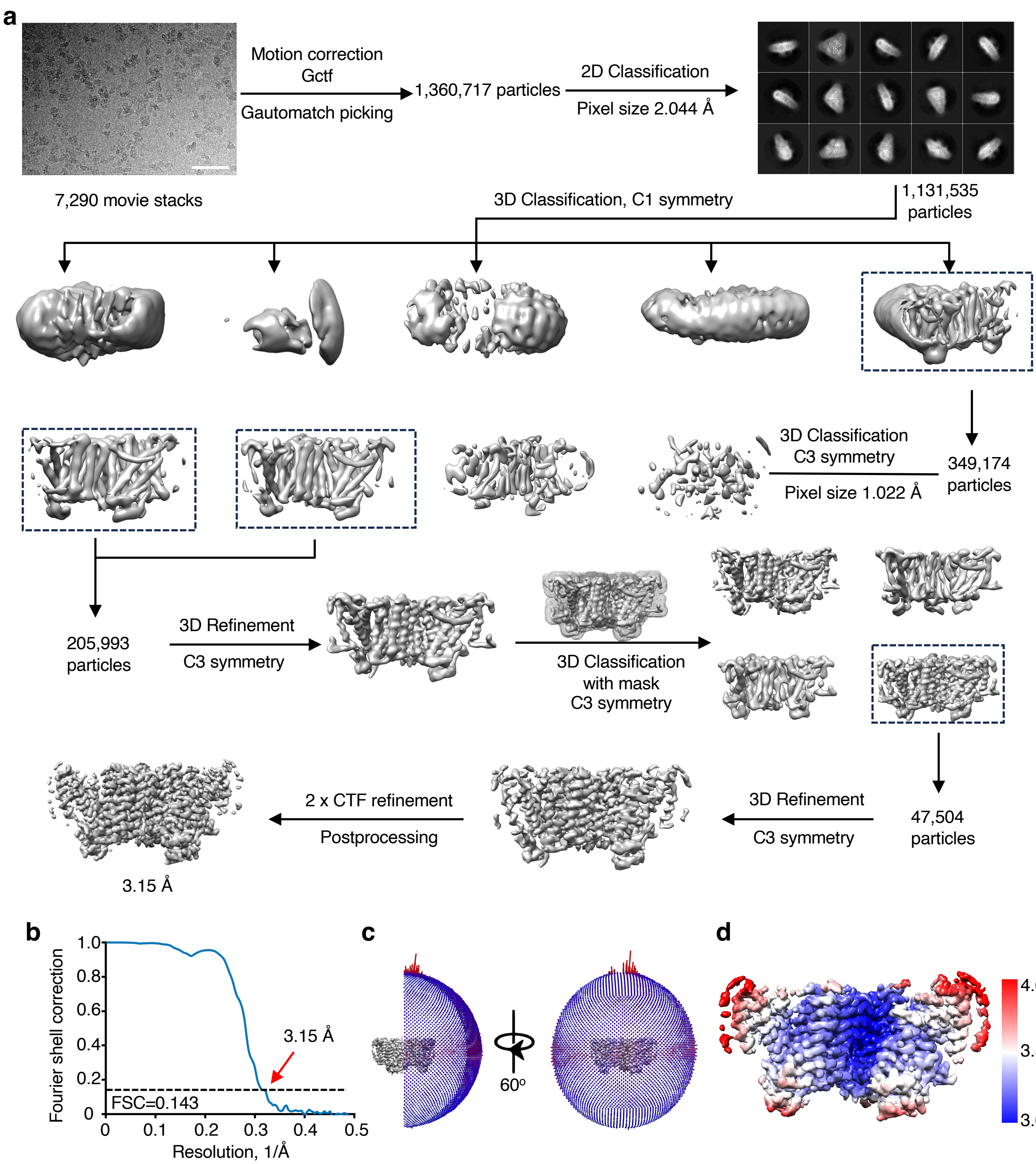

Supplementary Fig. 4 Cryo-EM data processing of trimeric NCLX. **a** Workflow of the cryo-EM data processing. The scale bar in the representative micrograph corresponds to 500 Å. **b** Golden-standard Fourier shell correction (FSC) curve of the 3D reconstruction. **c** Angular distribution of particle projections of the 3D reconstruction. **d** Local resolution of the rat trimeric NCLX estimated using RELION.

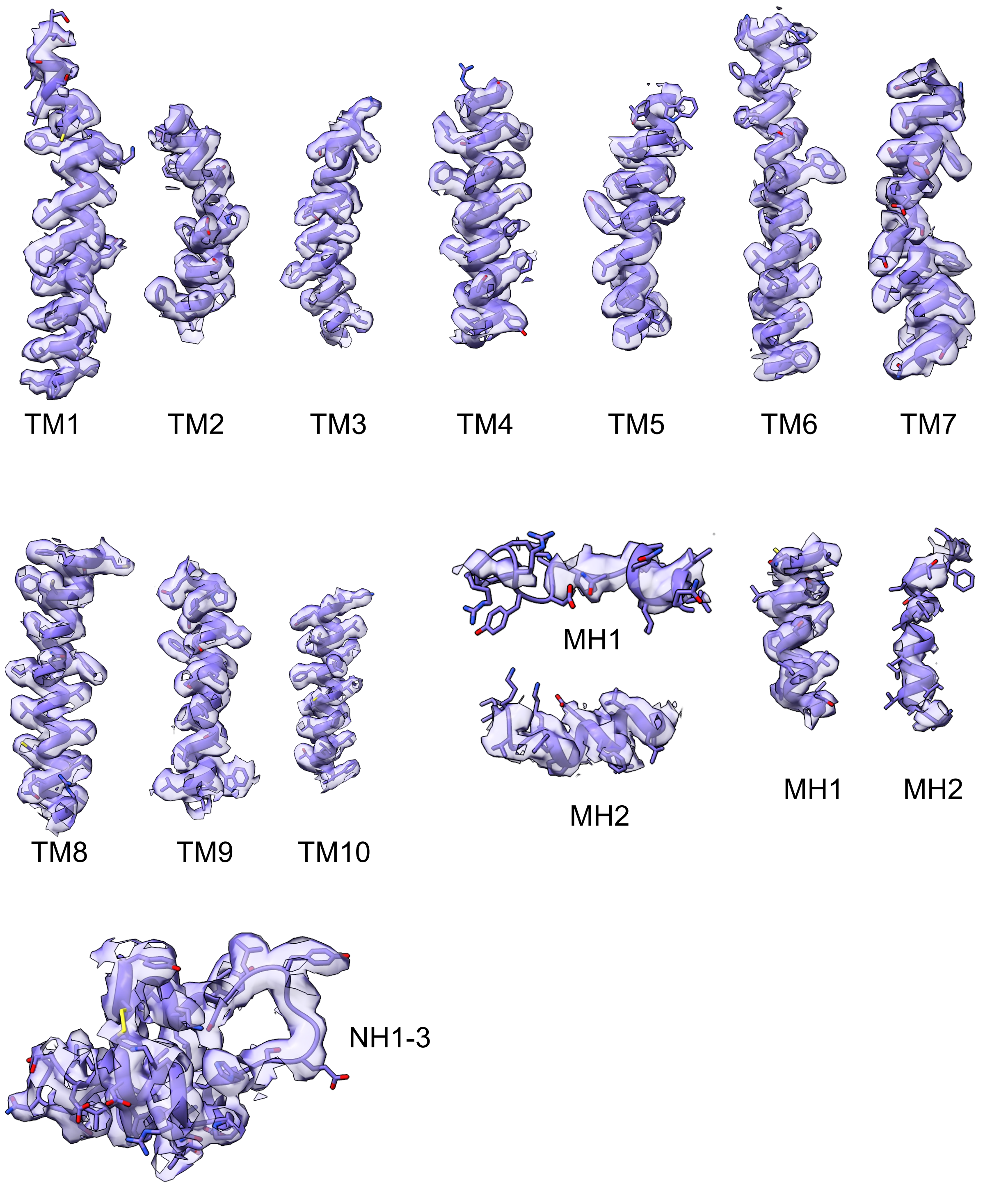

Supplementary Fig. 5 Density map of rat trimeric NCLX. Density maps of rat trimeric NCLX structure contoured at 6σ.

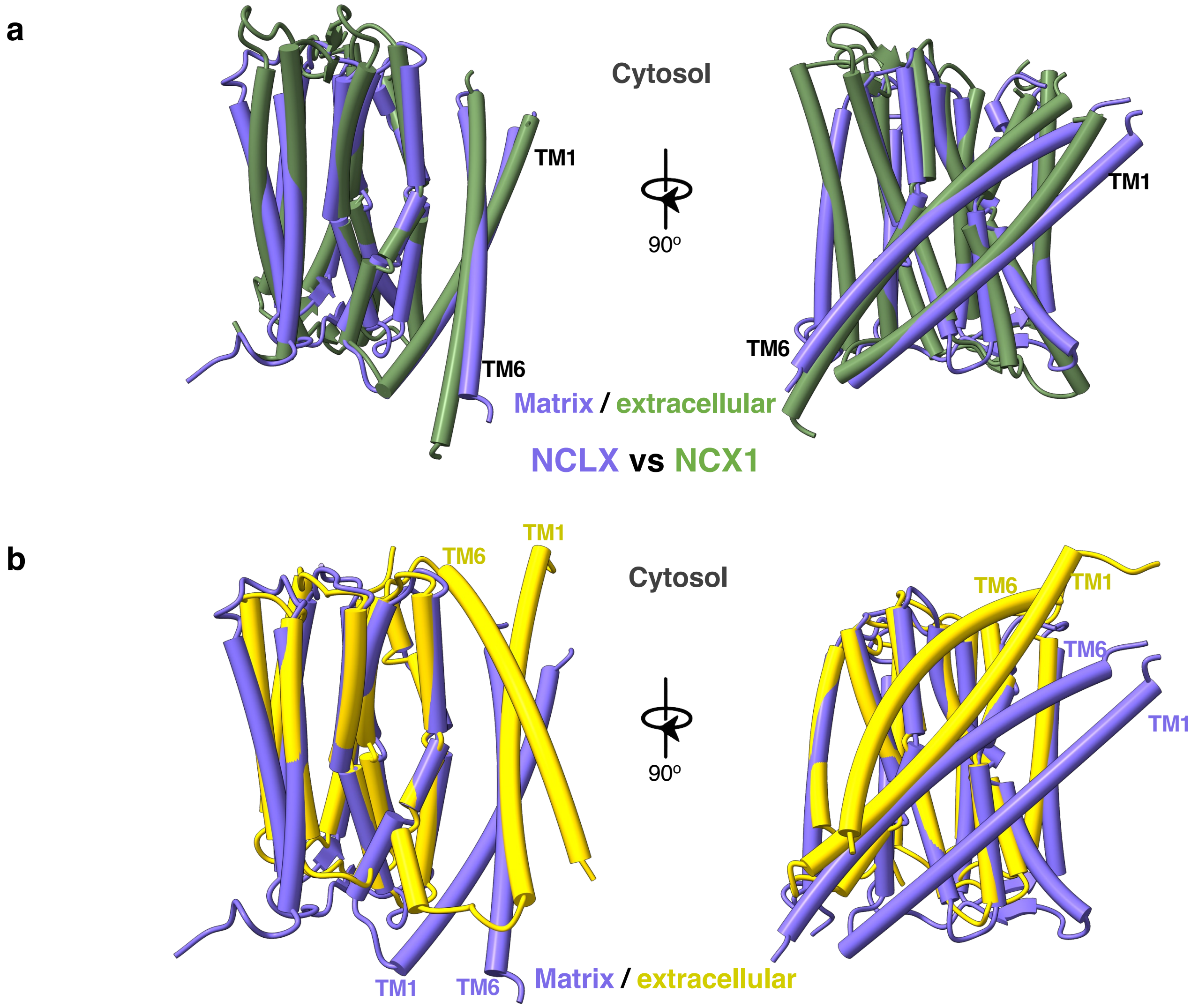

Supplementary Fig. 6 Structural comparison between NCLX and NCX. a Comparison of the transmembrane (TM) domains between trimeric NCLX (blue) and inward-facing human NCX1 (green; PDB 8SGJ). NCLX lacks the cytosolic regulatory domains present in NCX1, including the eXchanger Inhibitory Peptide (XIP) and the two calcium-binding domains (CBDs) responsible for Na^+-dependent inactivation[26]. We therefore limited our comparison to the TM regions that mediate ion exchange activity between NCLX and NCX1. **b** Comparison of the TM domains between trimeric NCLX (blue) and outward-facing NCX_Mj (yellow; PDB 3V5U). TMs 1 and 6 show the most pronounced structural differences between the two proteins. Note: NCLX is known to have its N- and C-termini facing the mitochondrial matrix[35]. When localized in plasma membrane upon overexpression in HEK cells, the C-terminus of NCLX has been shown to be exposed to the extracellular side[48]. Thus, the matrix side of NCLX is aligned with the extracellular side of NCXs in the structural comparison. This orientation for structural comparison at the TM region also ensures that the N- and C-terminal halves of NCLX are properly aligned with their counterparts in NCX.

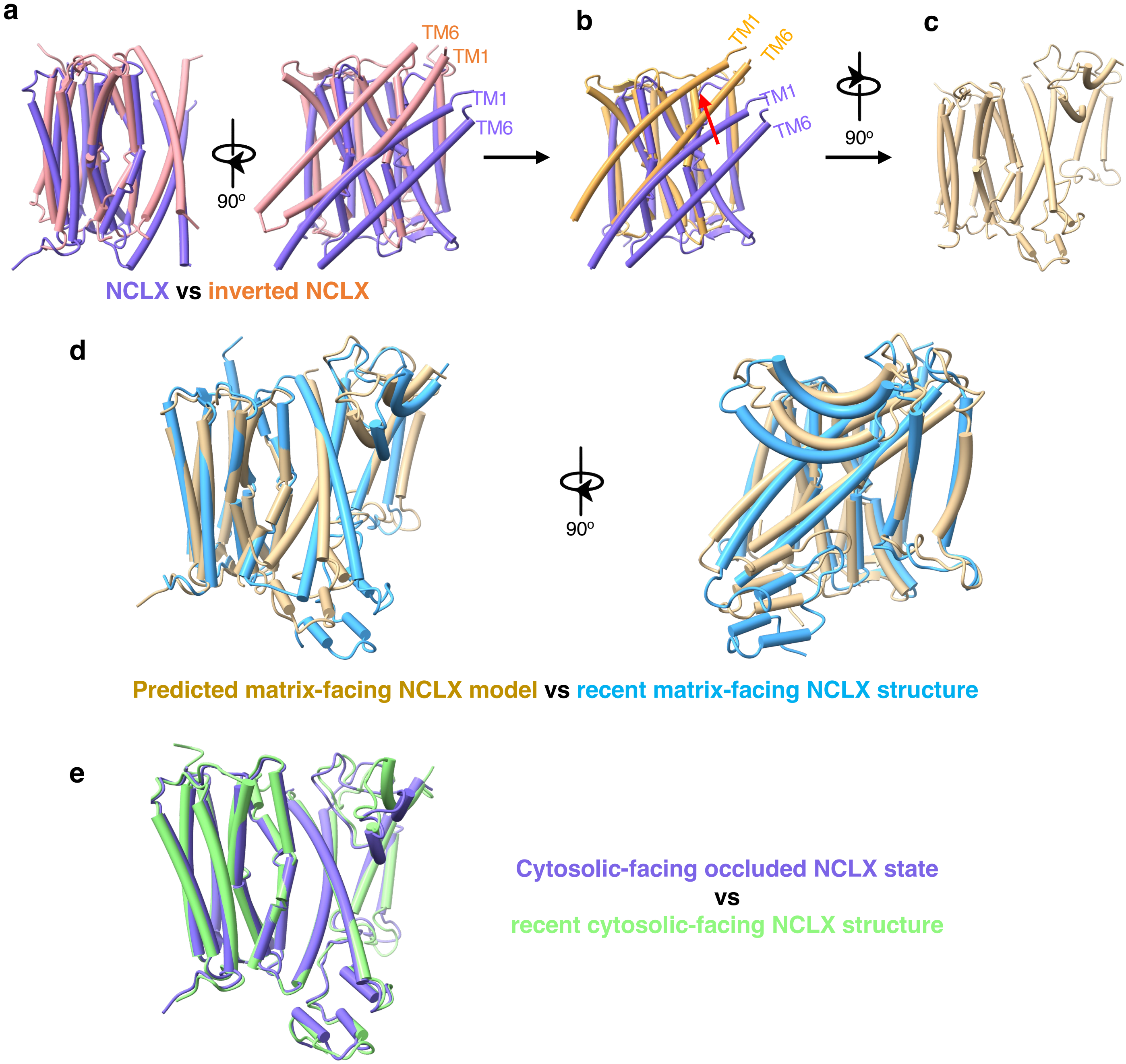

Supplementary Fig. 7 Generation of the matrix-facing NCLX model from the trimeric structure. a Step 1: Superimposition of NCLX (blue) with its inverted structure (pink). **b** Step 2: TMs 1 and 6 are repositioned to the locations of inverted TMs 6 and 1, respectively, as indicated by the red arrow. **c** Step 3: The matrix-facing model is generated by moving the auxiliary components together with TMs 1 and 6 as a rigid body. **d** Comparison of the proposed matrix-facing model (light gold) with the recently reported matrix-facing NCLX structure (cyan; PDB 9PS4). **e** Comparison of our cytosolic-facing occluded NCLX structure (blue) with the recently reported cytosolic-facing structure (green, PDB 9PS1).

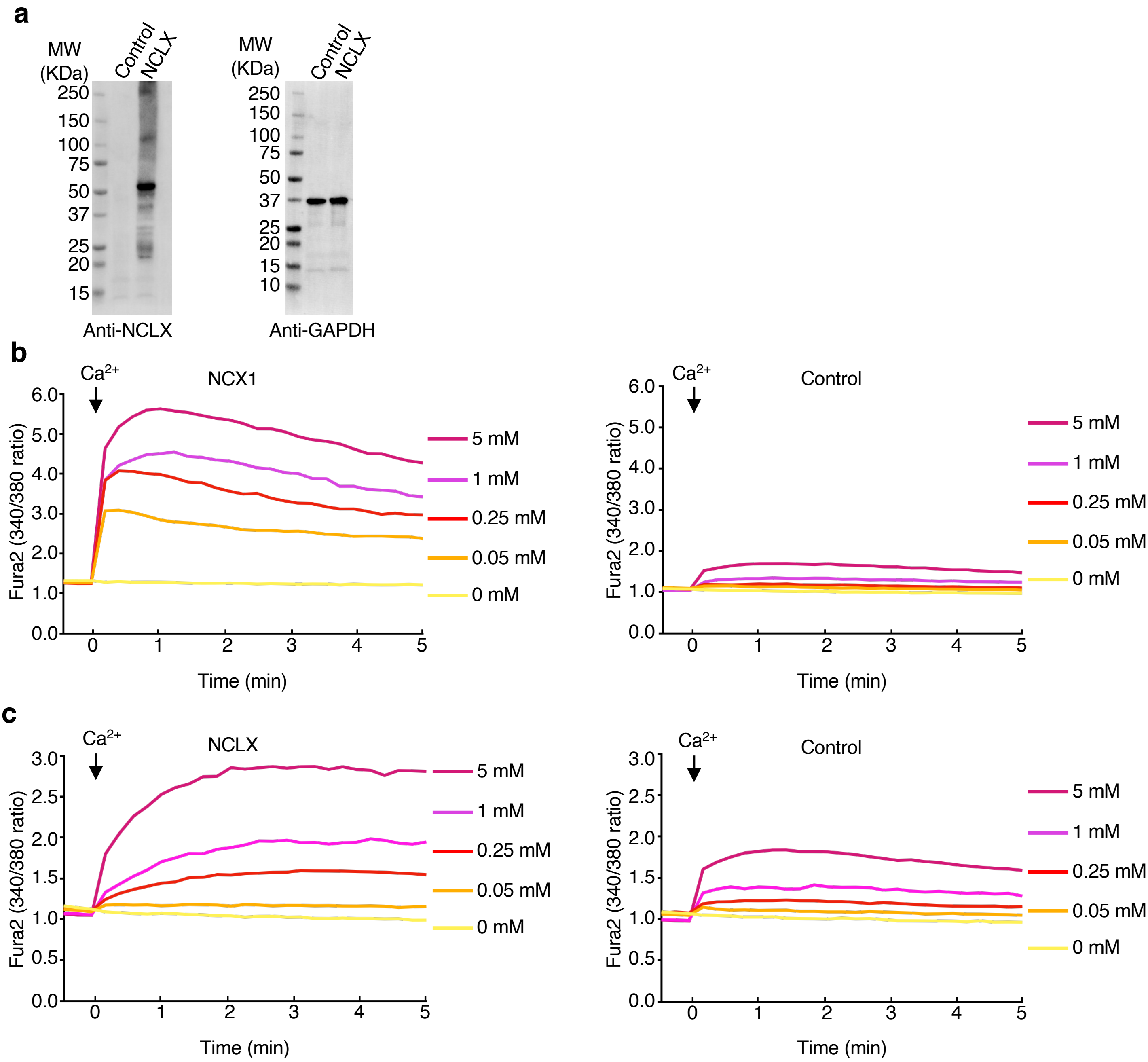

Supplementary Fig. 8 Sample preparation for Ca^{2+} uptake assays. a Surface expression of NCLX in HEK cells used for the Ca^{2+} uptake assay. Biotinylated surface NCLX was detected using an anti-NCLX antibody. Cells transfected with an empty vector served as a control. GAPDH (Glyceraldehyde-3-phosphate dehydrogenase) was used as a loading control. **b** Raw traces of Ca^{2+} uptake in NCX1-expressing HEK cells with 150 mM extracellular $NMDG^+$ and varying external [Ca^{2+}] (left), and corresponding traces from control HEK cells transfected with empty vector (right). Data in Fig. 5a were obtained by subtracting the control traces from the raw traces. The same procedure was applied to all Ca^{2+} uptake measurements in exchanger-expressing HEK cells under various conditions shown in Fig. 5. **c** Raw sample traces of Ca^{2+} uptake in NCLX1-expressing HEK cells (left) and control cells (right).

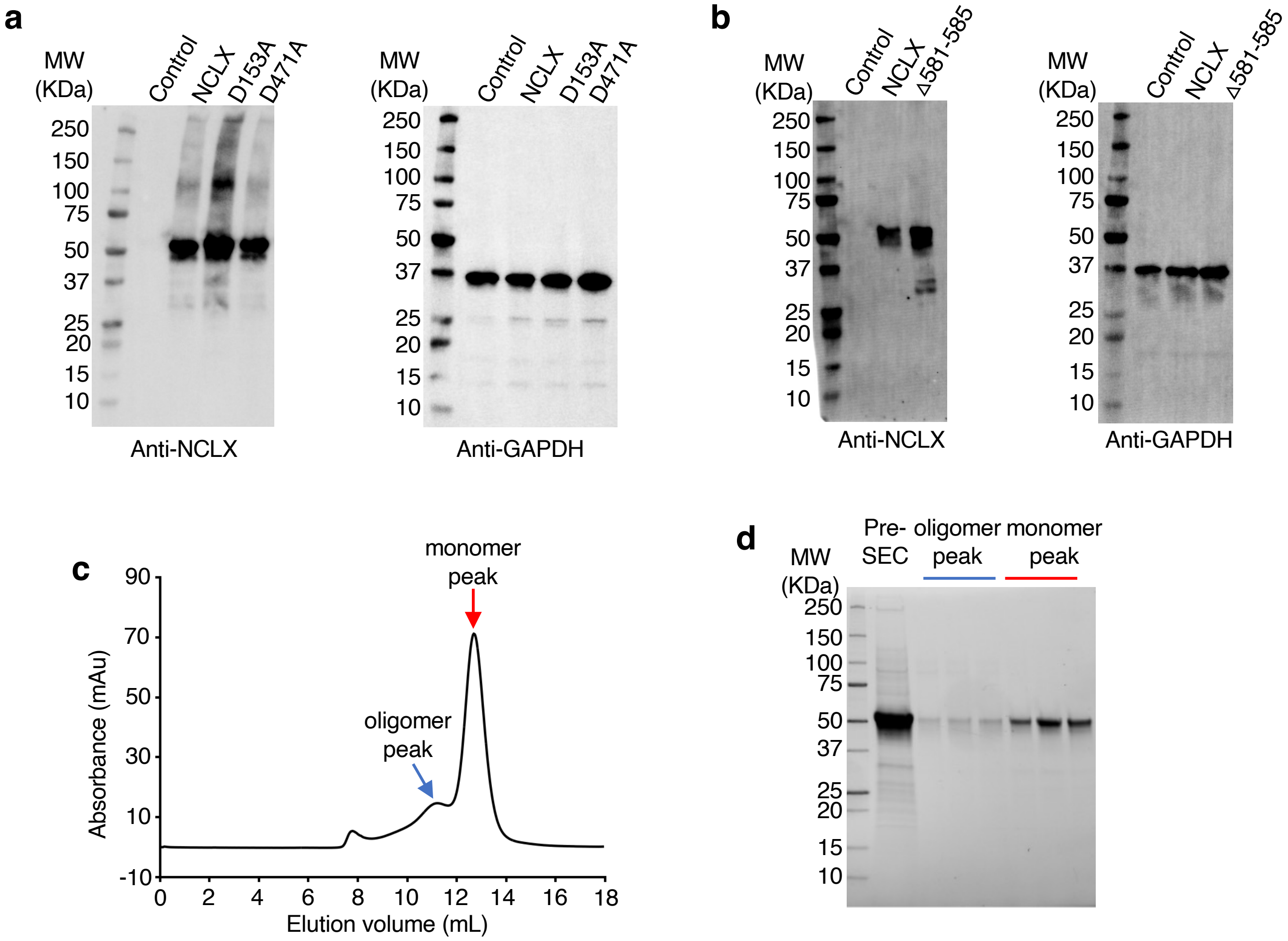

Supplementary Fig. 9 Biochemical analysis of rat NCLX mutants. **a, b** Surface expression of NCLX and its mutants in HEK cells used for the Ca^{2+} uptake assay. Total biotinylated surface NCLX and its mutants were detected using an anti-NCLX antibody. Cells transfected with an empty vector served as a control. GAPDH was used as a loading control. **c** The SEC profile of purified NCLX_Δ581-585 showing a much lower oligomeric peak. **d** SDS-PAGE of fractions collected from the two SEC peaks. Pre-SEC is the sample loaded for SEC analysis.

	NCLX_monomer (EMD-73804) (PDB 9Z4I)	NCLX_trimer (EMD-73805) (PDB 9Z4J)
**Data collection and processing**		
Magnification	165,000	130,000
Voltage (kV)	300	300
Electron exposure ($e^-/Å^2$)	60	60
Defocus range (μm)	-0.8 - -2.1	-0.8 - -2.1
Pixel size (Å/pixel)	0.743	0.511
Symmetry imposed	C1	C3
Initial particle images (no.)	4,616,468	1,360,717
Final particle images (no.)	336,224	47,504
Map resolution (Å)	3.62	3.15
FSC threshold	0.143	0.143
**Refinement**		
Initial model used (PDB code)		
Model resolution (Å)	3.65	3.00
FSC threshold	0.5	0.5
Model composition		
Non-hydrogen atoms	3,748	10,699
Protein residues	487	1383
Ligands	1: Na	3: Ca<br>1: Zn
B factor ($Å^2$)		
Protein	59.65	54.35
Ligand	30.00	52.48
R.m.s. deviations		
Bond length (Å)	0.003	0.005
Bond angles (°)	0.477	0.769
Validation		
MolProbity score	1.58	1.92
Clashscore	5.77	7.35
Rotamer outliers (%)	0.25	2.07
Ramachandran plot		
Outlier (%)	0	0
Allowed (%)	3.93	3.99
Favored (%)	96.07	96.01

Supplementary Table 1. Cryo-EM data collection and model statistics.

References

48. Cai, X. & Lytton, J. Molecular Cloning of a Sixth Member of the K^+-dependent Na^+/Ca^{2+} Exchanger Gene Family, NCKX6. *Journal of Biological Chemistry* **279**, 5867–5876 (2004).
