# Supplementary material for: Molecular mechanisms of mitochondrial Ca2+ exchanger NCLX
Source: Nat Commun. 2026 Jul 10;17:8523. doi: 10.1038/s41467-026-75483-x (PMC13483046; doi:10.1038/s41467-026-75483-x)
Supplement: Supplementary file 2 [file 41467_2026_75483_MOESM2_ESM.pdf]

nature portfolio

Corresponding author(s): Youxing Jiang

Last updated by author(s): Jun 11, 2026

Reporting Summary

Nature Portfolio wishes to improve the reproducibility of the work that we publish. This form provides structure for consistency and transparency in reporting. For further information on Nature Portfolio policies, see our Editorial Policies and the Editorial Policy Checklist.

Statistics

For all statistical analyses, confirm that the following items are present in the figure legend, table legend, main text, or Methods section.

n/a	Confirmed	
☐	☒	The exact sample size (*n*) for each experimental group/condition, given as a discrete number and unit of measurement
☐	☒	A statement on whether measurements were taken from distinct samples or whether the same sample was measured repeatedly
☐	☒	The statistical test(s) used AND whether they are one- or two-sided<br>*Only common tests should be described solely by name; describe more complex techniques in the Methods section.*
☒	☐	A description of all covariates tested
☒	☐	A description of any assumptions or corrections, such as tests of normality and adjustment for multiple comparisons
☐	☒	A full description of the statistical parameters including central tendency (e.g. means) or other basic estimates (e.g. regression coefficient) AND variation (e.g. standard deviation) or associated estimates of uncertainty (e.g. confidence intervals)
☐	☒	For null hypothesis testing, the test statistic (e.g. *F*, *t*, *r*) with confidence intervals, effect sizes, degrees of freedom and *P* value noted<br>*Give P values as exact values whenever suitable.*
☒	☐	For Bayesian analysis, information on the choice of priors and Markov chain Monte Carlo settings
☒	☐	For hierarchical and complex designs, identification of the appropriate level for tests and full reporting of outcomes
☒	☐	Estimates of effect sizes (e.g. Cohen's *d*, Pearson's *r*), indicating how they were calculated

Our web collection on statistics for biologists contains articles on many of the points above.

Software and code

Policy information about availability of computer code

Data collection	SerialEM 3.8, SpectraMax M6 spectrophotofluorometer
Data analysis	Relion 3.1, Relion 5.0, WinCoot 0.9.6, Chimera 1.16, Phenix 1.20.1, Pymol 3.1.1, ChimeraX 1.6.1, Microsoft Excel 16.77.1, Graphpad Prism 10

For manuscripts utilizing custom algorithms or software that are central to the research but not yet described in published literature, software must be made available to editors and reviewers. We strongly encourage code deposition in a community repository (e.g. GitHub). See the Nature Portfolio guidelines for submitting code & software for further information.

Data

Policy information about availability of data

All manuscripts must include a data availability statement. This statement should provide the following information, where applicable:

- Accession codes, unique identifiers, or web links for publicly available datasets
- A description of any restrictions on data availability
- For clinical datasets or third party data, please ensure that the statement adheres to our policy

The coordinates and the Cryo-EM maps are deposited in the Protein Data Bank (PDB) and Electron Microscopy Data Bank (EMDB) with accession codes: 9Z4I [http://doi.org/10.2210/pdb9Z4I/pdb] and EMD-73804 [https://www.ebi.ac.uk/pdbe/entry/emdb/EMD-73804] for rat monomeric NCLX, 9Z4J [http://doi.org/10.2210/pdb9Z4J/pdb] and EMD-73805 (https://www.ebi.ac.uk/pdbe/entry/emdb/EMD-73805] for rat trimeric NCLX. Atomic coordinates previously published and used for comparisons in this study are deposited in PDB under session numbers 8SGJ [http://doi.org/10.2210/pdb8SGJ/pdb] (human NCX1 in inward-facing conformation); 3V5U [http://doi.org/10.2210/pdb3V5U/pdb] (NCX_Mj in outward -facing conformation); 9PS4 [http://doi.org/10.2210/pdb9PS4/pdb] (rat NCLX in matrix-facing

conformation); 9PS1 [http://doi.org/10.2210/pdb9PS1/pdb] (rat NCLX in cytosolic-facing conformation). All the raw data of the cell-based calcium uptake assay and the relative statistical analysis are provided within the Source Data file. All materials used in this study, such as plasmids, can be obtained from the corresponding author upon request. Source data are provided with this paper.

Research involving human participants, their data, or biological material

Policy information about studies with human participants or human data. See also policy information about sex, gender (identity/presentation), and sexual orientation and race, ethnicity and racism.

Reporting on sex and gender	N/A
Reporting on race, ethnicity, or other socially relevant groupings	N/A
Population characteristics	N/A
Recruitment	N/A
Ethics oversight	N/A

Note that full information on the approval of the study protocol must also be provided in the manuscript.

Field-specific reporting

Please select the one below that is the best fit for your research. If you are not sure, read the appropriate sections before making your selection.

[x] Life sciences [ ] Behavioural & social sciences [ ] Ecological, evolutionary & environmental sciences

For a reference copy of the document with all sections, see nature.com/documents/nr-reporting-summary-flat.pdf

Life sciences study design

All studies must disclose on these points even when the disclosure is negative.

Sample size	The data size for cryo-EM was determined by the availability of the microscope time and the particle density on the grids. Sufficient cryo-EM datasets were collected to achieve the reported map resolution, which is sufficient for model building.
Data exclusions	CryoEM data processing involved removing poor-quality or damaged particles to achieve high resolution maps through pre-established standard data classification procedures.
Replication	For functional analysis, each experiment was reproduced at least four times. Experimental findings were reliably reproduced.
Randomization	Randomization was not necessary as the independent variables to be tested were sufficient for the functional interpretations within this study.
Blinding	All experiments are not blind. Blinding is not necessary for the purposes of structural determination. For functional analysis, blinding was not necessary due to the quantitative nature of the experiment.

Reporting for specific materials, systems and methods

We require information from authors about some types of materials, experimental systems and methods used in many studies. Here, indicate whether each material, system or method listed is relevant to your study. If you are not sure if a list item applies to your research, read the appropriate section before selecting a response.

Materials & experimental systems

n/a	Involved in the study	
[ ]	[x]	Antibodies
[ ]	[x]	Eukaryotic cell lines
[x]	[ ]	Palaeontology and archaeology
[x]	[ ]	Animals and other organisms
[x]	[ ]	Clinical data
[x]	[ ]	Dual use research of concern
[x]	[ ]	Plants

Methods

n/a	Involved in the study	
[x]	[ ]	ChIP-seq
[x]	[ ]	Flow cytometry
[x]	[ ]	MRI-based neuroimaging

Antibodies

Antibodies used	Anti-NCLX, invitrogen, Cat No. PA5-114330, Lot No. ZE4322922B, 1:1000 dilution<br>Anti-GAPDH, invitrogen, Cat No. 39-8600, Lot No. ZI403559, 1:1000 dilution<br>Goat anti-Mouse, invitrogen, Cat No. A21057, Lot No. 2491365, 1:5000 dilution<br>Goat anti-Rabbit, life technologies, Cat No. A21076, Lot No. 1655809, 1:5000 dilution
Validation	The primary antibodies were validated by the vendor.

Eukaryotic cell lines

Policy information about cell lines and Sex and Gender in Research

Cell line source(s)	Human embryonic kidney cells (HEK293S GnTI-) were purchased from ATCC and Sf9 insect cells were purchased from Thermo Fisher Scientific
Authentication	No further authentication was performed for commercially available cell lines
Mycoplasma contamination	Cell lines were not tested for mycoplasma contamination
Commonly misidentified lines (See ICLAC register)	None

Plants

Seed stocks	N/A
Novel plant genotypes	N/A
Authentication	N/A
