# Supplementary material for: Molecular mechanisms of mitochondrial Ca2+ exchanger NCLX
Source: Nat Commun. 2026 Jul 10;17:8523. doi: 10.1038/s41467-026-75483-x (PMC13483046; doi:10.1038/s41467-026-75483-x)
Supplement: Supplementary file 3 [file 41467_2026_75483_MOESM3_ESM.pdf]

nature portfolio

Peer Review File

Molecular mechanisms of mitochondrial Ca^{2+} exchanger NCLX

Corresponding Author: Professor Youxing Jiang

This file contains all reviewer reports in order by version, followed by all author rebuttals in order by version.

Version 0:

Reviewer comments:

Reviewer #1

(Remarks to the Author)
The study by Zhang et al. makes a significant contribution to our understanding of the mechanism of the mitochondrial sodium-calcium exchanger (NCLX), a key player in Ca^{2+} signaling and mitochondrial function.

While high-resolution structures of NCLX in multiple conformations, including outward- and inward-facing states, were recently reported (Nature 646, 1272–1280, 2025), Zhang et al. present new cytosolic-facing structures, providing critical insight into a local yet essential conformational transition in presence of transported ions, Ca^{2+} and Na^+. Specifically, the work captures the transition between the occluded and open states of the transporter, a structural change previously shown to be critical for ion recognition and specificity in homologous Na^+/Ca^{2+} exchangers (e.g., NCX_Mj; Liao et al., NSMB 2016; Marinelli et al., PNAS 2024). In addition, this study confirms the overall structural features of NCLX reported in recent work.

Another key contribution of this study addresses the functional specificity of NCLX, a topic that remains debated. While NCLX is generally considered a Na^+/Ca^{2+} exchanger, previous studies showed it can also transport Li^+, and data from Fan et al. (Nature 646, 1272–1280, 2025) suggested it might function as a Ca^{2+}/H^+ exchanger. Functional experiments in this study demonstrate that transport can be coupled to Li^+, Na^+, K^+ and , raising the possibility that NCLX acts as a relatively unspecific cation/Ca^{2+} antiporter.

The work also presents the structure of monomeric NCLX, showing that it retains the same fold as the trimeric form, which may represent the physiologically dominant assembly. The structure further suggests that NCLX can bind Na^+ in the central site which can also be occupied by Ca^{2+}.

Overall, this study provides important functional and structural insights into NCLX, and the work is very well executed and presented. I strongly recommend publication in Nature Communications, subject to a few minor corrections.

I suggest that the authors slightly expand the conclusion to discuss in more detail both the structural and functional results of their work in comparison with previous functional studies and the recently reported structures. Emphasizing both the differences and the specific contributions of this study would strengthen the manuscript.

I suggest that the authors discuss in more detail the observed effects of Li^+, Na^+, and K^+ on Ca^{2+} uptake. Specifically, while these ions may be transported, it cannot be excluded that some of them merely bind to the transporter without being translocated, potentially acting as blockers.

Finally, please verify the Zn^{2+} coordination; one would expect coordination with the nitrogen atoms of HIS residues (see, e.g., PDB ID 2SOD).

Reviewer #2

(Remarks to the Author)
NCLX is essential for mitochondrial Ca^{2+} homeostasis and cellular metabolism. This research presents cryo-EM structures of rat NCLX in cytosolic-facing occluded and open states, revealing a conserved alternating-access mechanism mediated by

sliding-door motions of TMs 1 and 6. Unlike canonical NCXs, NCLX lacks key Na^+-binding residues and functions as a non-selective cation/Ca^{2+} exchanger utilizing Na^+, K^+, Li^+, and potentially protons. These findings provide a structural framework for understanding mitochondrial Ca^{2+} signaling. Additionally, several major and minor concerns should be addressed to strengthen the study.

Major concerns:

1. The authors claim to have identified a zinc-binding site in NCLX, modeling the trimer-interface metal as Zn^{2+} based solely on three His581 residues and the general statement that mitochondria are Zn-rich. However, no direct evidence is provided to confirm that this density corresponds to Zn^{2+} rather than an artifact (e.g., C3 symmetry noise) or any other metal ion. Was Zn^{2+} demonstrated to be present in native cardiac or liver mitochondria at concentrations sufficient to support stable binding to His581 in vivo? Do the authors have data showing that Zn^{2+} chelation (e.g., with TPEN) affects oligomer stability or Ca^{2+} transport activity in situ? Furthermore, the cryo-EM density corresponding to the histidine cluster should be shown in a zoomed-in view, and it would be critical to examine whether mutations of this cluster impair the transporter function of NCLX.
2. The authors mentioned that TM9 and TM10 are involved in the formation of the NCLX trimer. However, unassigned density is observed between these helices that cannot be attributed to NCLX. What is the identity of this density? Does it contribute to trimer assembly or modulate NCLX function? Additionally, residues 312–318 show severe steric clashes in the trimer density map, and the loop regions 313–323 and 378–390 lack convincing supporting density. We recommend performing further structural optimization to resolve these clashes and further optimizing the local density or refining the atomic model at these positions.
3. The cryo-EM maps were sharpened with DeepEMhancer. What is the local resolution around the ion-binding pocket, and were the side-chain conformations of D153 and D471 validated by map-to-model FSC to ensure that the assigned Ca2+/Na+ densities are not over-interpreted from post-processed maps?
4. The reported resolution improvement of the trimeric NCLX reconstruction from ~3.96 Å to 3.15 Å is substantial. However, visual inspection of the final cryo-EM map reveals atypical side-chain density features, raising concerns about possible over-processing, particularly given the use of DeepEMenhancer. A more detailed description of the resolution improvement and map post-processing workflow is therefore needed to support the reliability of the final model.
5. The most critical concern is the potential non-selectivity of NCLX. Have the authors identified or analyzed potential binding sites for Li+, Na+, and K+ in the NCLX structures? Also, the assignment of Na+ at the SCa site in monomer NCLX lacks direct evidence. Have the author collected data of NCLX sample in normal NaCl buffer without EGTA, and further comparing these structures to definitively assign the ion identity? Have ion competition or binding assays been performed to confirm Na+ occupancy and its selectivity over Ca2+? Additionally, does Na^+ binding at SCa in the monomer induce conformational changes distinct from Ca^{2+} binding in the trimer?
6. A Ca^{2+} ion is assigned to the proposed SCa site in trimeric NCLX. However, direct experimental evidence demonstrating that this density corresponds specifically to Ca^{2+}, rather than another divalent cation or structured water, is currently lacking. The authors should clarify whether ion-specific validation experiments were performed, and functional validation through site-directed mutagenesis of coordinating residues (e.g., Asp153, Asp471) and Ca^{2+} binding or transport assays would further strengthen this assignment.
7. The manuscript characterizes NCLX as a non-selective cation/Ca^{2+} exchanger utilizing Na^+, K^+, Li^+, and H^+, and identifies TMs 9–10-mediated trimerization stabilized by Zn^{2+} binding. However, a recent study by Fan et al. (Nature 2025), employing NCLX knockout cell models and molecular dynamics simulations, robustly supports NCLX as a specific H^+/Ca^{2+} exchanger with no detectable Na^+ transport. Please reconcile this discrepancy.
8. The manuscript proposes that trimerization represents the native oligomeric state of NCLX, mediated by interactions involving TM9/10 and Zn^{2+}-coordinated His581, while the monomeric form is attributed to detergent-induced disruption. It would be important to clarify whether NCLX trimerization has been validated in a native membrane context. For example, have the authors assessed NCLX oligomerization using approaches such as FRET, co-immunoprecipitation, or size-exclusion chromatography of mitochondrial extracts under near-native conditions? In addition, the functional Ca^{2+} uptake assays were conducted using plasma membrane–localized NCLX in GnTI⁻ cells, which may not reflect its physiological environment or oligomeric state in the mitochondrial inner membrane. If trimerization represents the native and functionally relevant state, does the monomeric structure correspond to a detergent-induced artifact, or could it represent a physiologically relevant form?
9. Functional assays show that Li^+, Na^+, and K^+ all inhibit NCLX-mediated Ca^{2+} uptake, supporting its role as a non-selective cation/Ca^{2+} exchanger. However, the stoichiometry of exchange is noted as unresolved. Could the authors comment on whether multiple monovalent cations are transported per Ca^{2+}, and whether this might explain the slower kinetics?
10. The manuscript references recent studies suggesting that TMEM65, rather than NCLX, plays the determinant role in mitochondrial calcium regulation and export, yet the conclusion continues to emphasize NCLX as a critical regulator without further discussing potential functional redundancy between NCLX and TMEM65 in calcium signaling. Do the authors have evidence for physical or functional interactions between these two proteins? Would TMEM65 knockdown affect the function of NCLX? It is advisable to supplement this content in the discussion section.
11. The authors report that NCLX mediates Ca^{2+} exchange with “substantially slower transport kinetics than NCX”, yet the structural basis for this kinetic difference remains unclear. They propose that NCLX-specific auxiliary helices (MH3–4, NH1–3), which are partially embedded in the membrane and interact with TM1 and TM6, may restrict the sliding motion of these transmembrane segments. It would be helpful to clarify which specific residues in MH3/MH4 interact with TMs 1/6, how these interactions constrain the “sliding-door” motion during alternating access, and whether deletion these helices accelerate ion exchange. Could molecular dynamics simulations be employed to directly compare the conformational dynamics of these key regions between NCLX and NCX? The physiological significance of the slower transport rate could be discussed in the context of mitochondrial calcium signaling.

Minor concerns:

1. Line numbers should be added to the manuscript.
2. The abbreviations of Sint, Sext, and Smid are not defined in the manuscript or figure legends. The authors should define these terms at their first occurrence.
3. In main Figure 2c, the left and right panels clearly show the same view, however, the stick radius and sphere sizes appear inconsistent between the two panels. The authors should correct this issue.
4. Scale bars need to be added to the micrographs in Fig. S2 and S3.
5. The notation “2X CTF” should be changed to “2 × CTF”; please check the entire manuscript for consistent use of the multiplication sign.
6. The particle-orientation distribution plot in Fig. S3 appears mis-aligned and shows very few particles, please verify that it corresponds to the final reconstruction map.
7. I don't have specific experimental data about how the SEC fraction peak and gel image would change when purifying monomeric NCLX with 1 mM EGTA.
8. Page 5, line7, Fig. 1f should be cited.
9. The figure legend for Fig. S6b-c is mismatched, and the legend for Fig. S6d-e is missing.
10. The role of NH1-3 in conformational changes deserves discussion.
11. The legend of Fig. S3d should be corrected from monomeric NCLX to trimeric NCLX.
12. Page 7, line 19. What accounts for the localization of overexpressed NCLX to the plasma membrane in HEK cells instead of its native mitochondrial inner membrane?
13. Page 5, line 26. Does the more open cavity of NCLX permit coordination of divalent cations beyond Ca^{2+}?

Reviewer #3

(Remarks to the Author)
I co-reviewed this manuscript with one of the reviewers who provided the listed reports. This is part of the Nature Communications initiative to facilitate training in peer review and to provide appropriate recognition for Early Career Researchers who co-review manuscripts.

Reviewer #4

(Remarks to the Author)
In this study, the authors determined cryo-EM structures of rat NCLX in monomeric and trimeric forms, corresponding to the cytosolic-facing open and occluded conformational states, respectively. By comparison with plasma membrane Na^+-Ca^{2+} exchanger NCXs, the authors argue that the central transmembrane module of NCLX adopts an architectural framework conserved among NCX family exchangers; however, the absence of Na^+-binding residues renders the exchanger nonselective for monovalent cations during Ca^{2+} exchange. To further substantiate this idea, the authors performed cell-based functional assays, demonstrating that NCLX mediates slower Ca^{2+} uptake than NCX, and exhibits sensitivity not only to Na+, but also to Li^+, K^+, and H^+. Overall, the study is well designed, and the results are interesting and are likely to make a significant contribution to this field. However, several explanations are insufficient, and some of the interpretations appear to be too speculative. Given that structures of rat NCLX in distinct conformational states have been reported recently (Fan et al., Nature, 2025), a more thorough and rigorous comparative structural analysis is necessary. Specific comments are as follows.

Major
1. page 4, 2nd paragraph. “Considering the mitochondrial localization of NCLX, the matrix-facing side corresponds to the extracellular side of NCXs in our comparisons (Fig. S4).”. NCX in its forward mode extrudes cytosolic Ca^{2+} in exchange for extracellular Na^+, whereas NCLX in its forward mode extrudes matrix Ca^{2+} in exchange for cytosolic Na^+. This reviewer is not fully convinced that the present alignment is appropriate. Related to this, the human NCX1 structure (PDB ID: 8SGJ) demonstrated by the present authors captures a Na^+-dependent inactivated state, a recognized regulatory feature of NCX1, not of NCLX, that is proposed to lie outside the canonical Na^+/Ca^{2+} exchange cycle. In this context, what would the interpretation change if the NCLX structure were aligned with 8SGT, which represents a Ca^{2+}-bound, activated state of NCX1 that is permissive for transport?
2. page 4, last paragraph. “This arrangement suggests that these elements form part of a regulatory module with TMs 1 and 6 and are likely to move together as a rigid body during conformational cycling.”. Please show data supporting the role of these parts as a “regulatory module”.
3. page 5, 1st paragraph. Please provide supporting data or relevant literature, if available, regarding transition metal-mediated regulation of NCLX.
4. The authors argue that the ion density at S_{Ca} site in monomeric NCLX structure corresponds to Na+, as Ca^{2+} was chelated by EGTA. If this is the case, why is Na^+ not observed around the open cavity in trimeric NCLX structure, which corresponds to S_{int}, S_{mid}, and S_{ext} in NCX_Mj, even though Na^+ is the only monovalent cation present under this condition?
5. Please provide a more detailed discussion comparing the present structure with the reported NCLX structure in the cytosol-facing conformation (Fan et al., Nature, 2025).
6. Functional analyses. Please clarify the topology of NCLX in the plasma membrane of HEK cells. The authors evaluated the activities of NCX and NCLX in their “reverse mode”, i.e., Ca^{2+} influx from extracellular space to cytosol in exchange for cytosolic monovalent cations, possibly Na+. The slowing of Ca^{2+} entry by application of extracellular monovalent cations

does not necessarily mean that these cations are directly exchanged for intracellular Ca2+. Please provide direct evidence demonstrating that Ca^{2+} is exchanged for monovalent cations.
7. page 9. “We speculate that membrane-embedded elements closely associated with TMs 1 and 6, but absent in NCX, may restrict their mobility and consequently slow the exchange cycle”. It would be interesting to investigate potential functional changes resulting from the removal of mutation of these regions in NCLX.

Minor
page 12. “*in vitro*” rather than “*in vivo*”?

Version 1:

Reviewer comments:

Reviewer #1

(Remarks to the Author)
The authors have adequately addressed all major concerns, and I believe the manuscript is now ready for publication.

Reviewer #2

(Remarks to the Author)
The authors have addressed the previous comments by providing additional clarifications, experimental data, and supporting references. These revisions improve the overall clarity and presentation of the manuscript.

Minor concerns:
1. In supplementary Figures 1a and 1c, the horizontal axes share the same scale, but the overall figure dimensions differ substantially. The authors should unify the panel sizes and align the two panels properly.
2. The legend of Supplementary Figure 6c should be corrected from NCX1 to NCLX1.
3. In the response to major concerns 1, the left and right panels clearly show the same view, however, the displayed size of Zn2+ is inconsistent between the two panels. Additionally, the Supplementary Fig. 7a-b cited in the response should be corrected to Supplementary Fig. 7c.

Reviewer #3

(Remarks to the Author)
I co-reviewed this manuscript with one of the reviewers who provided the listed reports. This is part of the Nature Communications initiative to facilitate training in peer review and to provide appropriate recognition for Early Career Researchers who co-review manuscripts.

Reviewer #4

(Remarks to the Author)
I appreciate the authors' efforts in revising the manuscript. While it has been significantly improved in this revision, there are still a few areas where the explanations remain insufficient and require further clarification.

Major
1. Supplementary Fig. 4. This reviewer understands that the TM region of the Ca2+-bound, activated NCX1 structure (PDB ID: 8SGT) is poorly resolved and therefore cannot be used for precise analysis. However, as noted previously, the Na+-dependent inactivated state lies outside the canonical Na+/Ca2+ exchange cycle. That is, it is not part of the normal transport turnover. It should be referred to in the manuscript.

2. Ca2+ uptake assay. It would be helpful to present the uptake kinetics (or rates), in addition to the level of Ca2+ accumulation, especially for the mutants.

3. It is practically possible to record NCLX currents using the patch-clamp technique (PMID: 32354321).

Open Access This Peer Review File is licensed under a Creative Commons Attribution 4.0 International License, which permits use, sharing, adaptation, distribution and reproduction in any medium or format, as long as you give appropriate credit to the original author(s) and the source, provide a link to the Creative Commons license, and indicate if changes were made.
In cases where reviewers are anonymous, credit should be given to 'Anonymous Referee' and the source.
The images or other third party material in this Peer Review File are included in the article's Creative Commons license, unless indicated otherwise in a credit line to the material. If material is not included in the article's Creative Commons license and your intended use is not permitted by statutory regulation or exceeds the permitted use, you will need to obtain permission directly from the copyright holder.
To view a copy of this license, visit https://creativecommons.org/licenses/by/4.0/

Response to Reviewers

Reviewer #1:

The study by Zhang et al. makes a significant contribution to our understanding of the mechanism of the mitochondrial sodium-calcium exchanger (NCLX), a key player in Ca^{2+} signaling and mitochondrial function.

While high-resolution structures of NCLX in multiple conformations, including outward- and inward-facing states, were recently reported (Nature 646, 1272-1280, 2025), Zhang et al. present new cytosolic-facing structures, providing critical insight into a local yet essential conformational transition in presence of transported ions, Ca^{2+} and Na^{+}. Specifically, the work captures the transition between the occluded and open states of the transporter, a structural change previously shown to be critical for ion recognition and specificity in homologous Na^{+}/Ca^{2+} exchangers (e.g., NCX_Mj; Liao et al., NSMB 2016; Marinelli et al., PNAS 2024). In addition, this study confirms the overall structural features of NCLX reported in recent work.

Another key contribution of this study addresses the functional specificity of NCLX, a topic that remains debated. While NCLX is generally considered a Na^{+}/Ca^{2+} exchanger, previous studies showed it can also transport Li^{+}, and data from Fan et al. (Nature 646, 1272-1280, 2025) suggested it might function as a Ca^{2+}/H^{+} exchanger. Functional experiments in this study demonstrate that transport can be coupled to Li^{+}, Na^{+}, K^{+} and, raising the possibility that NCLX acts as a relatively unspecific cation/Ca^{2+} antiporter.

The work also presents the structure of monomeric NCLX, showing that it retains the same fold as the trimeric form, which may represent the physiologically dominant assembly. The structure further suggests that NCLX can bind Na^{+} in the central site which can also be occupied by Ca^{2+}.

Overall, this study provides important functional and structural insights into NCLX, and the work is very well executed and presented. I strongly recommend publication in Nature Communications, subject to a few minor corrections.

We appreciate reviewer #1's positive comments and constructive suggestions. The general comments raised by the reviewer are addressed as follows:

1. I suggest that the authors slightly expand the conclusion to discuss in more detail both the structural and functional results of their work in comparison with previous functional studies and the recently reported structures. Emphasizing both the differences and the specific contributions of this study would strengthen the manuscript.

As suggested, we have expanded the Discussion section in the revised manuscript to provide a more comprehensive comparison of our structural and functional findings with those reported in previous studies.

2. I suggest that the authors discuss in more detail the observed effects of Li^+, Na^+, and K^+ on Ca^{2+} uptake. Specifically, while these ions may be transported, it cannot be excluded that some of them merely bind to the transporter without being translocated, potentially acting as blockers.

All our functional assays were performed alongside NCX1, a well-characterized Na^+-dependent Ca^{2+} exchanger, under identical experimental conditions. Although our cell-based competition assays cannot fully exclude the possibility that these monovalent ions act as blockers rather than participating directly in ion exchange, several observations support their role as counter-exchanging ions. First, Ca^{2+} uptake is not completely abolished even at high concentrations of monovalent cations, arguing against simple competitive blockade and instead suggesting that reduced uptake reflects an increased inward gradient of counterions opposing Ca^{2+} influx. Second, in Na^+-selective NCX1, only Na^+ suppresses Ca^{2+} uptake, whereas in NCLX, Li^+, Na^+, and K^+ produce comparable inhibitory effects. This makes it unlikely that Na^+ acts as the exchange ion in NCX1 while these same monovalent cations function as blockers in NCLX. Third, the high structural similarity between NCX1 and NCLX, particularly within the α-repeat regions that mediate ion exchange, supports a conserved alternating-access mechanism. The absence of specific Na^+-binding residues in NCLX likely permits broader access of monovalent cations to the ion-binding pocket. Within this framework, monovalent cations that enter the pocket from one side at high concentration are expected to be released to the opposite side during conformational cycling, thereby functioning as counterions in Ca^{2+} transport. We have incorporated this discussion into the revised Discussion section.

3. Finally, please verify the Zn^{2+} coordination; one would expect coordination with the nitrogen atoms of HIS residues (see, e.g., PDB ID 2SOD).

We have corrected the orientation of the His581 imidazole ring to ensure proper coordination of Zn^{2+} via its nitrogen atom.

Reviewer #2:

NCLX is essential for mitochondrial Ca^{2+} homeostasis and cellular metabolism. This research presents cryo-EM structures of rat NCLX in cytosolic-facing occluded and open states, revealing a conserved alternating-access mechanism mediated by sliding-door motions of TMs 1 and 6. Unlike canonical NCXs, NCLX lacks key Na^+-binding residues and functions as a non-selective cation/Ca^{2+} exchanger utilizing Na^+, K^+, Li^+, and potentially protons. These findings provide a structural framework for understanding mitochondrial Ca^{2+} signaling. Additionally, several major and minor concerns should be addressed to strengthen the study.

We appreciate reviewer #2's insightful comments and constructive suggestions. The points raised are addressed below:

Major concerns:

1. The authors claim to have identified a zinc-binding site in NCLX, modeling the trimer-interface metal as Zn^{2+} based solely on three His581 residues and the general statement that mitochondria are Zn-rich. However, no direct evidence is provided to confirm that this density corresponds to Zn^{2+} rather than an artifact (e.g., C3 symmetry noise) or any other metal ion. Was Zn^{2+} demonstrated to be present in native cardiac or liver mitochondria at concentrations sufficient to support stable binding to His581 in vivo? Do the authors have data showing that Zn^{2+} chelation (e.g., with TPEN) affects oligomer stability or Ca^{2+} transport activity in situ? Furthermore, the cryo-EM density corresponding to the histidine cluster should be shown in a zoomed-in view, and it would be critical to examine whether mutations of this cluster impair the transporter function of NCLX.

Regarding the identity of the metal ion, we cannot definitively conclude that it is Zn^{2+}. The density map reveals a strong non-protein density located beneath the histidine cluster, positioned to interact with the side chains of the surrounding histidine residues. The geometry of this density (Fig. 1e) is consistent with histidine-mediated coordination of Zn^{2+}, as observed in some known zinc-binding proteins (Wolfgand Maret, J Inorg Biochem, 2012, PMID: 22196021), and Zn^{2+} is among several divalent metal ions present in the mitochondrial matrix (Liu et al., Int J Physiol Pathophysiol Pharmacol, 2016, PMID: 27186321). Based on these considerations, we tentatively modeled the density as a Zn^{2+} ion. However, we cannot exclude the possibility that it represents another metal ion. This clarification has been incorporated into the revised manuscript.

To rule out the possibility that the observed density arises from C3 symmetry–related artifacts, we reprocessed the dataset and performed 3D reconstruction without imposing symmetry, yielding a map at 3.4 Å resolution. In this reconstruction, the strong density corresponding to the putative ion remains clearly visible and lies beneath the histidine cluster, supporting the presence of a bound metal ion. For comparison, here we provide zoomed-in views of the histidine cluster in trimeric NCLX derived from reconstructions processed with and without symmetry. In both maps, the density modeled as a Zn^{2+} ion is shown as a grey mesh contoured at 11σ. A zoomed-in view of the histidine cluster, along with its corresponding density, has been shown in Fig. 1f.

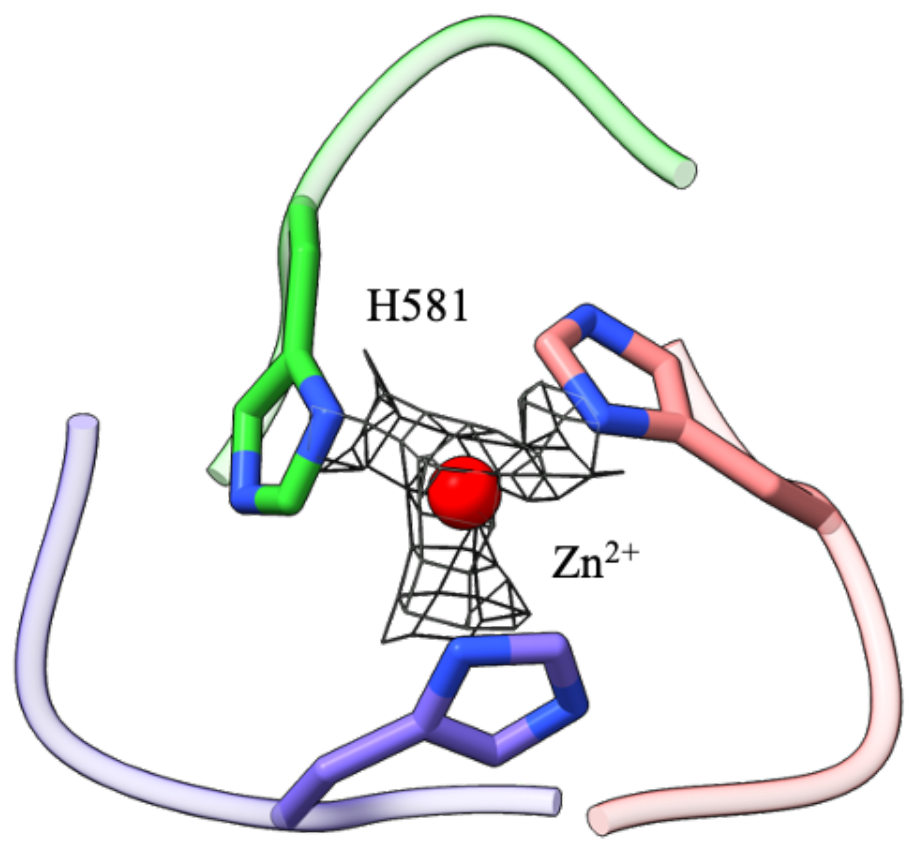

Reconstruction with C3 symmetry

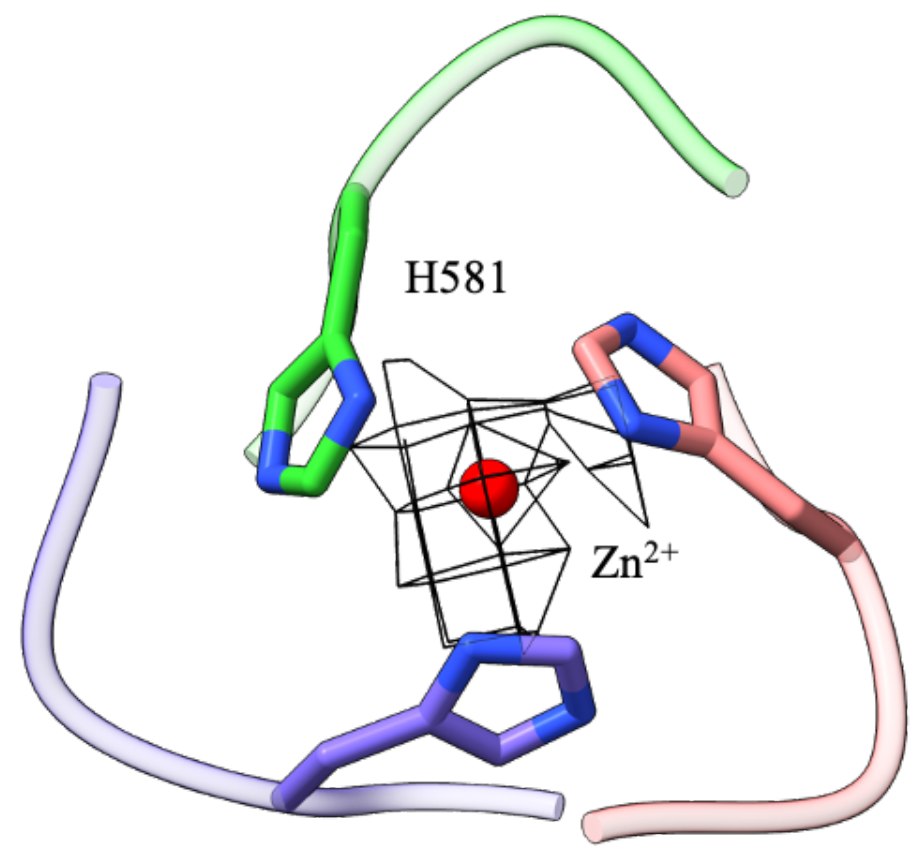

Reconstruction with C1 symmetry

To evaluate the potential role of the putative Zn^{2+}-binding site, we generated an NCLX mutant lacking residues 581-585. Size-exclusion chromatography revealed a reduced trimer-to-monomer ratio compared with the wild-type protein (Supplementary Fig. 7a-b), indicating that the C-terminal histidine cluster contributes to trimer assembly. Functional analysis of the truncated mutant showed a marked reduction in activity relative to wild-type NCLX (Fig. 5h). Together, these results demonstrate that the C-terminal histidine cluster is critical for both trimer assembly and NCLX function. These findings have been incorporated into the revised manuscript (Fig. 5 and Supplementary Fig. 7).

2. <u>The authors mentioned that TM9 and TM10 are involved in the formation of the NCLX trimer. However, unassigned density is observed between these helices that cannot be attributed to NCLX. What is the identity of this density? Does it contribute to trimer assembly or modulate NCLX function? Additionally, residues 312-318 show severe steric clashes in the trimer density map, and the loop regions 313-323 and 378-390 lack convincing supporting density. We recommend performing further structural optimization to resolve these clashes and further optimizing the local density or refining the atomic model at these positions.</u>

We observed an additional density at the interface between TM9 and the adjacent TM10 on the matrix-facing side. Based on its overall shape and location, this density is most likely attributable to a lipid molecule co-purified with the protein during extraction. However, given the high heterogeneity of cellular lipids and the lack of sufficient structural features in the density, we were unable to assign this identity with confidence and therefore left it unmodeled in the final structure. TM9 and TM10 primarily form part of the structural scaffold and are involved in trimer assembly. The presence of a lipid molecule at this interface may contribute to stabilizing the trimeric assembly; however, without definitive identification, its functional role cannot be directly assessed.

Due to the absence of resolvable density for residues 378-390, this segment was omitted from the final model. In contrast, residues 313-323 are better resolved in the monomeric structure. Structural alignment indicates that the monomeric and trimeric NCLX structures are highly similar, with only subtle differences in a portion of the α-repeat helix. Based on this high degree of similarity, we remodeled this region in the trimer using the monomeric structure as a reference and subsequently refined it to resolve steric clashes.

3. The cryo-EM maps were sharpened with DeepEMhancer. What is the local resolution around the ion-binding pocket, and were the side-chain conformations of D153 and D471 validated by map-to-model FSC to ensure that the assigned Ca^{2+}/Na^{+} densities are not over-interpreted from post-processed maps?

The trimeric NCLX map was sharpened using DeepEMhancer, whereas the monomeric map was processed using RELION post-processing. DeepEMhancer was used to improve map interpretability for visualization and model building; however, all structural interpretations and resolution estimates were based on the original reconstruction. As shown below, the local resolution in the ion-binding pocket of trimeric NCLX is around 3.0 - 3.2 Å, while that of monomeric NCLX is around 3.5 - 3.7 Å. The density maps are displayed at a contour level of 4σ.

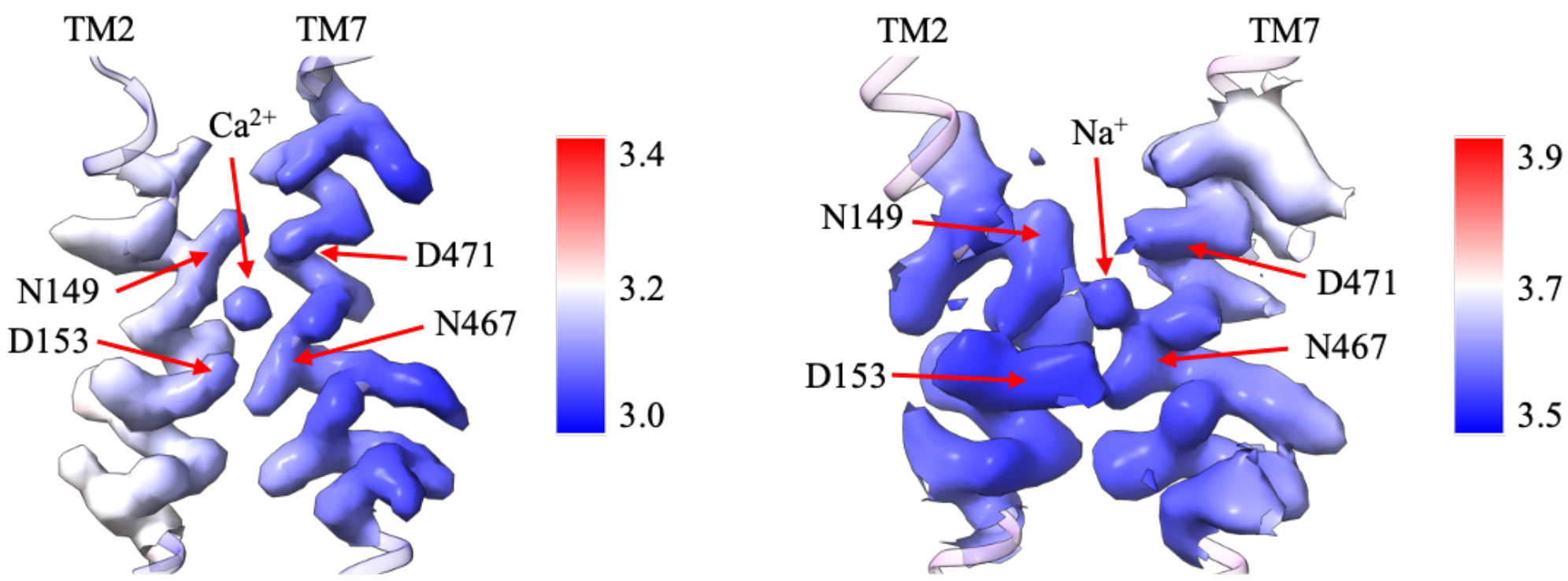

Trimeric NCLX

Monomeric NCLX

The model-to-map agreement was evaluated during real-space refinement in Phenix. For trimeric NCLX, the real-space correlation coefficients are 0.775 for residue D153 and 0.802 for D471; for monomeric NCLX, the corresponding values are 0.835 and 0.704, respectively. These values indicate that both residues are well supported by the cryo-EM density and their modeled conformations are reliable. The map-to-model FSC curves of trimeric and monomeric NCLX are shown below.

Map-to-model FSC of trimeric NCLX

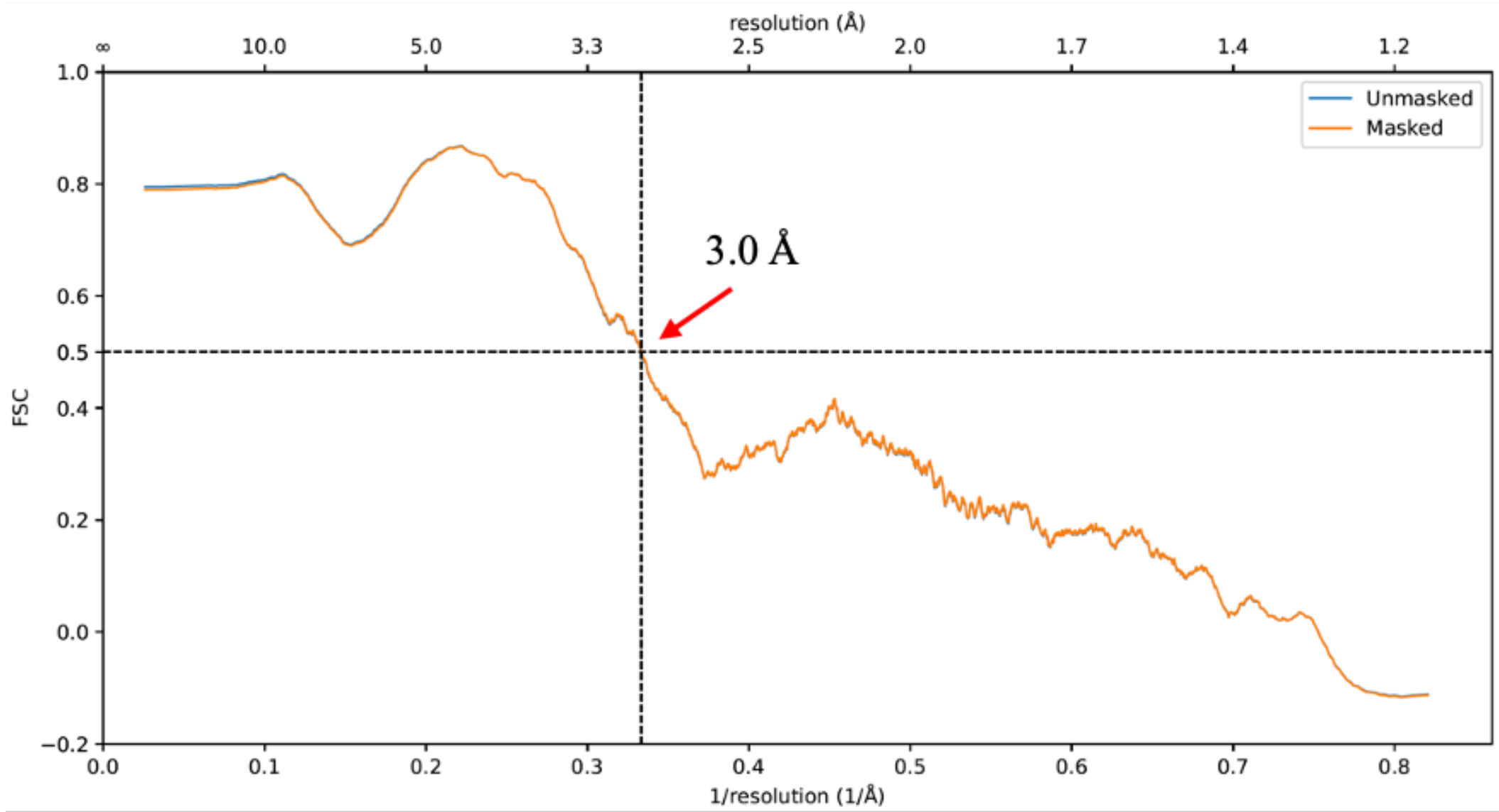

Map-to-model FSC of monomeric NCLX

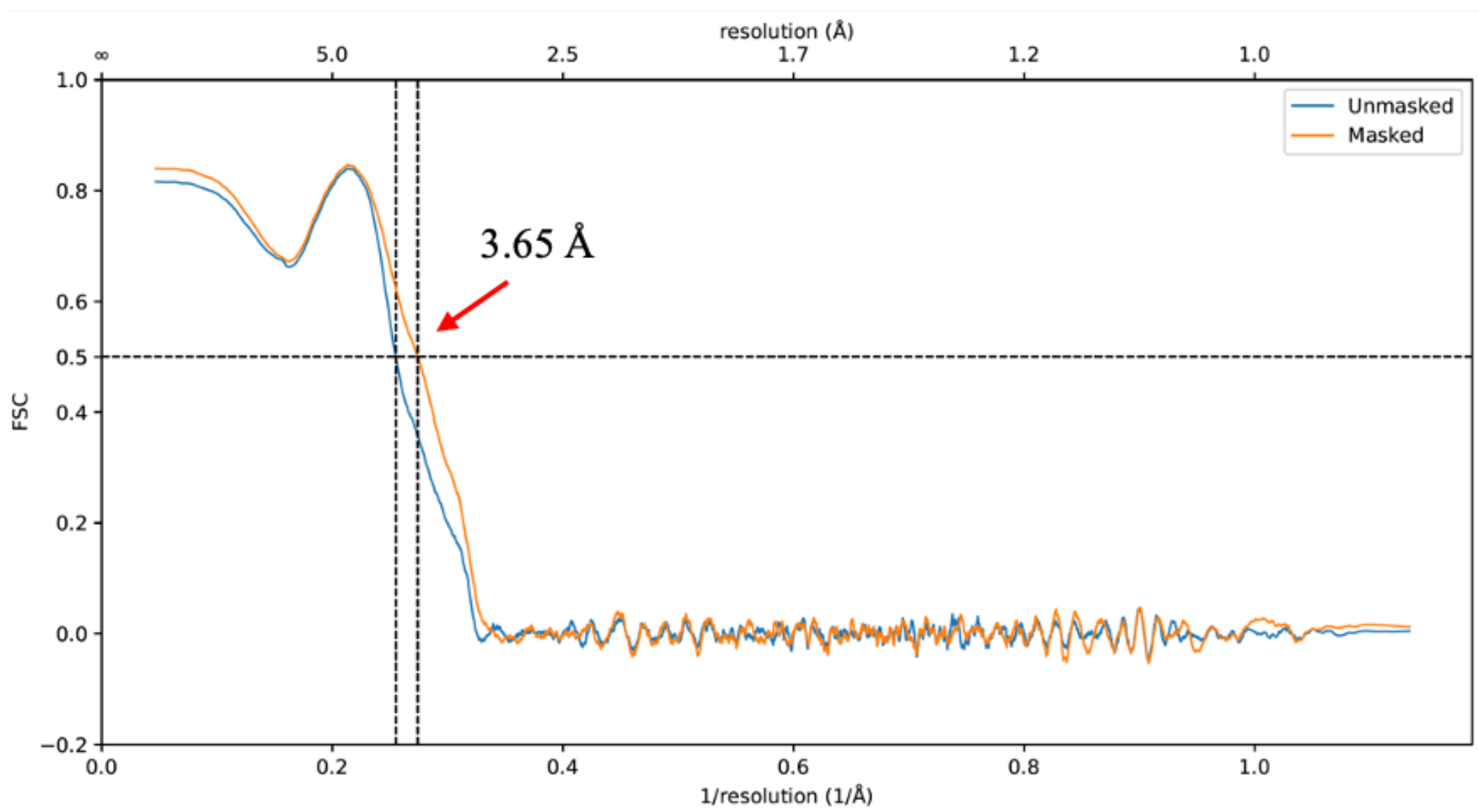

4. <u>The reported resolution improvement of the trimeric NCLX reconstruction from ~3.96 Å to 3.15 Å is substantial. However, visual inspection of the final cryo-EM map reveals atypical side-chain density features, raising concerns about possible over-processing, particularly given the use of DeepEMenhancer. A more detailed description of the resolution improvement and map post-processing workflow is therefore needed to support the reliability of the final model.</u>

The 3.96 Å map corresponds to the output from 3D auto-refinement (no masking), whereas the final 3.15 Å map was obtained after post-processing with a mask covering the protein density.

Specifically, applying a similar mask to the 3.96 Å auto-refined map and performing post-processing yielded a 3.32 Å reconstruction, as shown below. Subsequent CTF refinement provided a modest additional improvement of 0.18 Å. To avoid confusion, we have removed the labelled resolution values from the intermediate steps in Supplementary Fig. 2 and Supplementary Fig. 3.

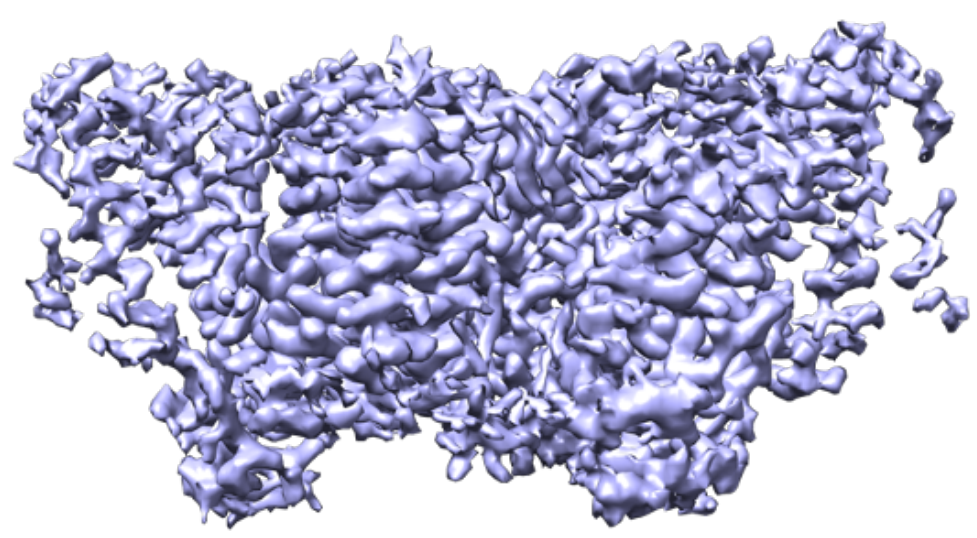

Post-processed map derived from the 3.96 Å auto-refinement

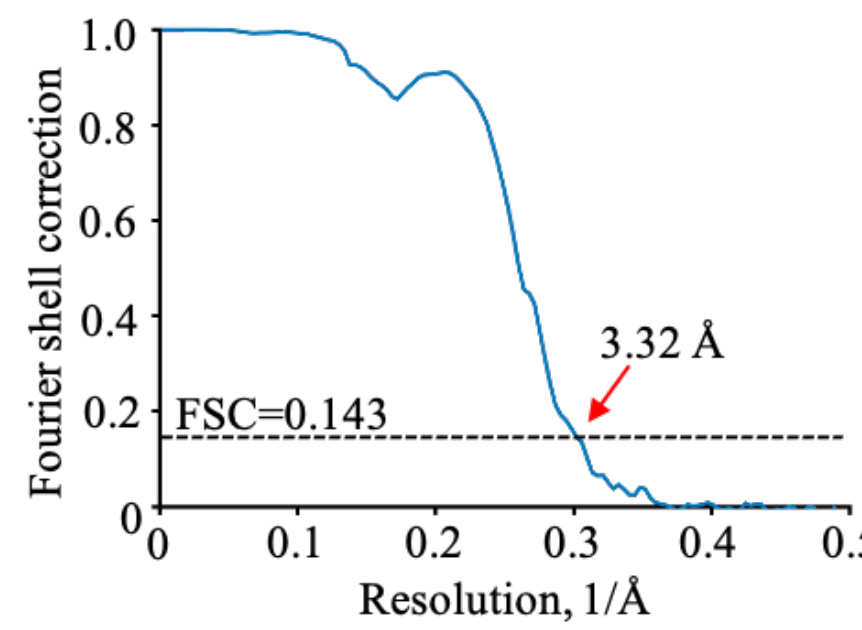

FSC curve of the post-processed map

Here are the details about the data-processing used to improve the resolution. After 3D classification, the particles from the good class were selected for 3D auto-refinement. To further improve the high-resolution features of the reconstruction, the refined particle set was subjected to CTF refinement. In this step, we first corrected microscope-induced aberrations at the micrograph level, including beam tilt, trefoil and 4th order aberrations, followed by the estimation of anisotropic magnification. Subsequently, per-particle defocus values were refined to account for local variations across individual particles. We performed two cycles of these CTF refinements to ensure the parameters are converged. A schematic summarizing all steps of the CTF refinement procedure is provided below.

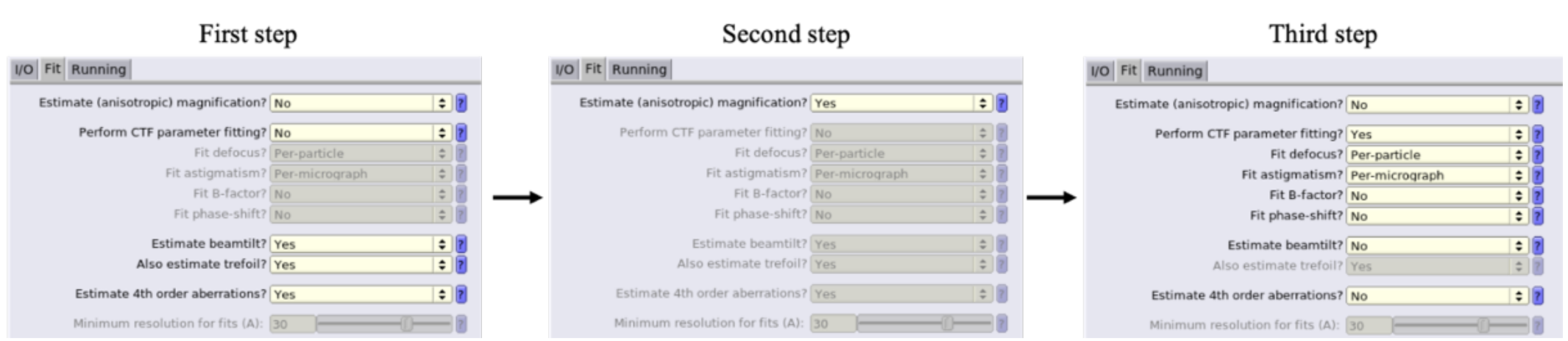

The half maps from the 3D reconstruction, together with a mask covering the protein density, were imported for post-processing to improve the interpretability of the cryo-EM density map and enhance high-resolution features by B-factor sharpening. Post-processing was performed using the default parameters, yielding a final map at 3.15 Å resolution.

For local resolution estimation, half maps from the 3D reconstruction and the corresponding protein mask were used. The B-factor applied for local resolution estimation was set to the value determined during the post-processing step.

Regarding the use of DeepEMhancer, we provide the following detailed explanation. Post-processing sharpens the map using a global B-factor, which can sometimes lead to over-sharpening of noisy regions, appearing as discontinuous density. DeepEMhancer is a deep-learning-based tool that performs implicit local sharpening of cryo-EM volumes, enhancing different regions according to their local signal quality. The neural network suppresses background noise and

solvent density, producing maps with improved contrast and cleaner features which facilitates manual model building and interpretation of structural details. The network was trained on a large set of experimentally determined cryo-EM maps.

For visualization and model-building guidance, we applied DeepEMhancer using half maps from the 3D reconstruction. As shown below, we present the cryo-EM density maps, including the local-resolution-filtered map (Relion_locres_filtered) generated by RELION during local resolution estimation, as well as the DeepEMhancer-processed map (DeepEMhancer sharpened map). Both maps are additionally displayed with coloring according to the estimated local resolution. The DeepEMhancer-processed map improves map interpretability without introducing apparent over-processing, producing a cleaner density for structural inspection. During model building, all residues in the final model were carefully validated by comparing features observed in both the DeepEMhancer-processed map and the standard post-processed map, ensuring that model construction was not solely dependent on the DeepEMhancer-enhanced density.

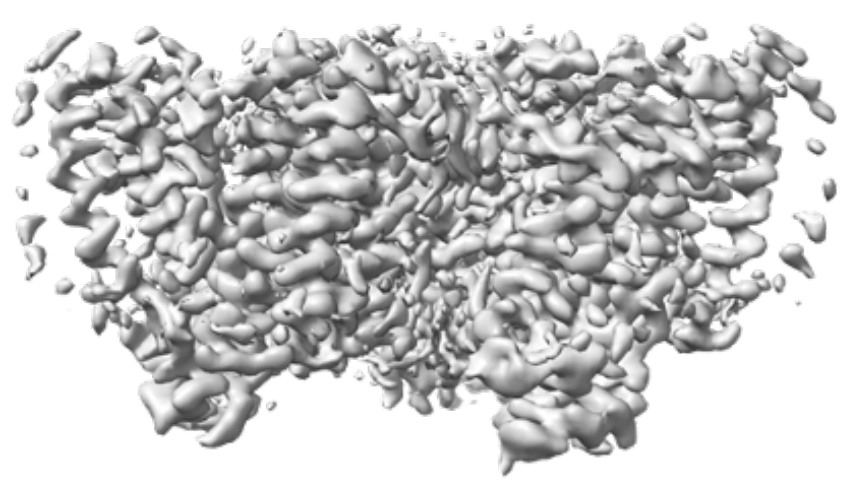
Relion_locres_filtered map

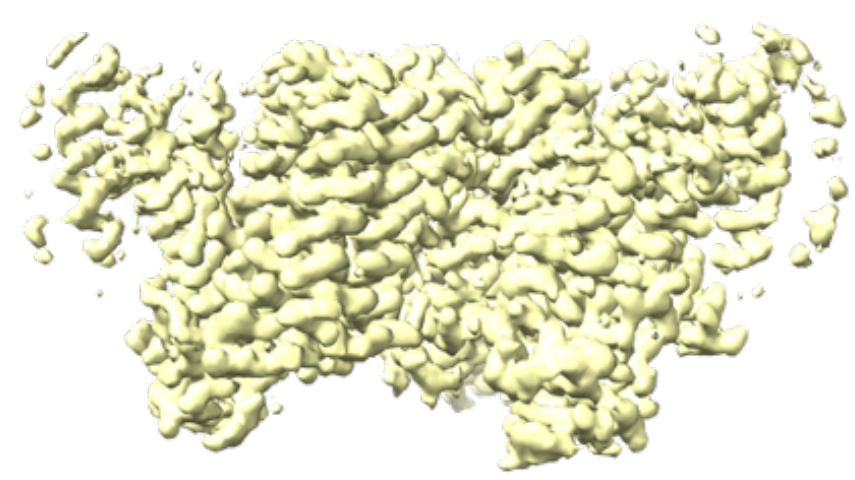
DeepEMhancer sharpened map

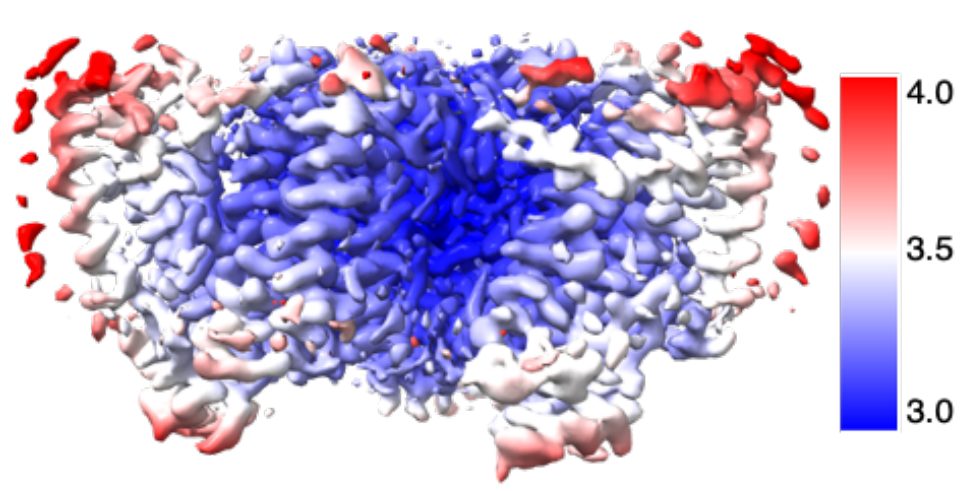

Relion_locres_filtered map
colored by local resolution

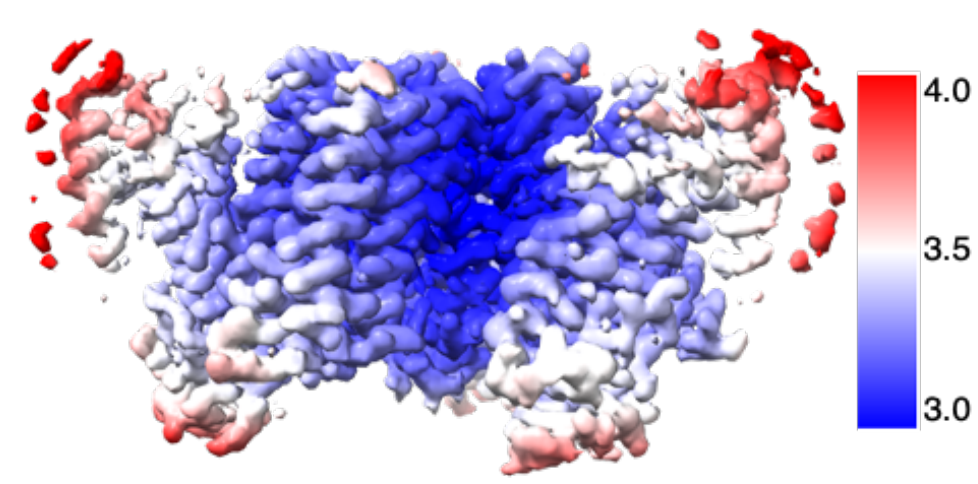

DeepEMhancer sharpened map
colored by local resolution

The descriptions of the resolution improvement and map post-processing workflow were provided in the Methods section of the revision.

5. <u>The most critical concern is the potential non-selectivity of NCLX. Have the authors identified or analyzed potential binding sites for Li^+, Na^+, and K^+ in the NCLX structures? Also, the assignment of Na^+ at the S_{Ca} site in monomer NCLX lacks direct evidence. Have the author collected data of NCLX sample in normal NaCl buffer without EGTA, and further comparing these structures to definitively assign the ion identity? Have ion competition or binding assays been performed to confirm Na^+ occupancy and its selectivity over Ca^{2+}? Additionally, does Na^+ binding at S_{Ca} in the monomer induce conformational changes distinct from Ca^{2+} binding in the trimer?</u>

The conclusion that NCLX functions as a non-selective monovalent cation/Ca^{2+} exchanger is supported by numerous previous studies in the field. This conclusion is further supported by our functional assays comparing NCLX with the Na^+-selective Ca^{2+} exchanger NCX1. Structurally, aside from the S_{Ca} site, we did not identify additional potential monovalent cation-binding sites in NCLX, consistent with the recent structural study by Fan et al. (Fan et al., Nature 2025, PMID: 40931067). In our study, we performed a detailed structural comparison between NCLX and NCX_Mj at the ion exchange pocket to provide insights into NCLX selectivity (Fig. 2c-d). Previous X-ray studies of NCX_Mj identified three ion-binding sites: S_{int} and S_{ext} for Na^+ binding, and S_{Ca} for high-affinity Ca^{2+} binding, with Na^+ able to occupy S_{Ca} in the absence of Ca^{2+}. The structural conservation of S_{Ca} in NCLX suggests that it likewise serves as a high-affinity Ca^{2+} site. Supporting this, functional assays added in the revised manuscript (Fig. 5g) show that mutations at S_{Ca} site markedly reduce Ca^{2+} uptake. In contrast, key sidechains responsible for Na^+ binding at S_{int} and S_{ext} in NCX_Mj are replaced in NCLX - S236 at S_{int} becomes Gly, and S77 at S_{ext} becomes Ala. These substitutions likely explain the loss of Na^+ selectivity in NCLX, enabling other monovalent cations to access the ion-binding pocket and function as exchange ions for Ca^{2+}.

Regarding ion assignment in our structures, the trimeric NCLX sample was prepared in a nominal NaCl buffer without EGTA, containing micromolar free Ca^{2+}. Given the high Ca^{2+} affinity at S_{Ca}, we assign the observed density at this site to Ca^{2+}. The monomeric NCLX was prepared in NaCl buffer supplemented with EGTA, which chelates trace Ca^{2+}, leaving Na^+ as the predominant monovalent cation. Accordingly, the density at the S_{Ca} site in the monomeric structure is assigned to Na^+. Notably, Na^+ binding at S_{Ca} triggers an outward movement of the TM2ab segment from the core transmembrane module, disrupting the Ca^{2+}-coordinating backbone carbonyl of Asn149 at S_{Ca}. The rationale for this ion assignment is detailed in the manuscript.

Due to the multi-ion exchange mechanism of Ca^{2+} exchangers, along with changes in Ca^{2+} affinity depending on counter ion concentrations and exchanger conformation, it is challenging to analyze ion competition, occupancy, and affinity using conventional biophysical tools. To our knowledge, electrophysiological recordings with giant patches have been the only method used to characterize these parameters in NCX. However, patch-clamp recording is not feasible for NCLX because of its much slower exchange activity.

6. A Ca^{2+} ion is assigned to the proposed S_{Ca} site in trimeric NCLX. However, direct experimental evidence demonstrating that this density corresponds specifically to Ca^{2+}, rather than another divalent cation or structured water, is currently lacking. The authors should clarify whether ion-specific validation experiments were performed, and functional validation through site-directed mutagenesis of coordinating residues (e.g., Asp153, Asp471) and Ca^{2+} binding or transport assays would further strengthen this assignment.

The residues surrounding the S_{Ca} site are highly conserved between NCLX and other well-characterized Na^+/Ca^{2+} exchangers, including NCX1 and the archaeal NCX_Mj. Previous studies have demonstrated that S_{Ca} exhibits much higher affinity for Ca^{2+} than for Na^+ (Iwaki et al., FEBS, 2020, PMID: 32056381; Miura & Kimura, JGP, 1989, PMID: 2549177; Liao et al., NSMB, 2016, PMID: 27183196). In the trimeric NCLX sample, the protein was prepared in nominally Ca^{2+}-free buffer, which typically contains micromolar Ca^{2+} (John et al., JGP, 2018, PMID: 29301861). Considering both structural conservation and experimental conditions, the non-protein density

observed at S_{Ca} was modeled as a Ca^{2+} ion. To validate this assignment functionally, we generated D153A and D471A mutants. Both mutants exhibited a marked reduction in NCLX activity (included in the revised manuscript - Fig. 5g), supporting the essential role of these acidic residues and confirming that the density at S_{Ca} most likely corresponds to Ca^{2+}.

7. The manuscript characterizes NCLX as a non-selective cation/Ca^{2+} exchanger utilizing Na^+, K^+, Li^+, and H^+, and identifies TMs 9-10-mediated trimerization stabilized by Zn^{2+} binding. However, a recent study by Fan et al. (Nature 2025), employing NCLX knockout cell models and molecular dynamics simulations, robustly supports NCLX as a specific H^+/Ca^{2+} exchanger with no detectable Na^+ transport. Please reconcile this discrepancy.

NCLX knockout cell models in Fan's study suggested that NCLX may not play a major role in mitochondrial Ca^{2+} efflux. Two recent studies propose that TMEM65, rather than NCLX, mediates rapid mitochondrial Ca^{2+} extrusion. However, this conclusion remains debated, and we have briefly discussed these findings in the revised Discussion section. Notably, the MD simulations in Fan's study support Ca^{2+} binding at S_{Ca}, consistent with our conclusions.

The primary discrepancy between our study and Fan et al. lies in functional characterization: our assays indicate that NCLX operates as a non-selective cation/Ca^{2+} exchanger, including H^+/Ca^{2+} exchange, whereas their results suggest exclusive H^+/Ca^{2+} exchange activity. Given the distinct experimental approaches, this difference is not easily reconciled. Importantly, all our assays were conducted alongside NCX1, a well-characterized Na^+-selective Ca^{2+} exchanger, under identical conditions. Under these conditions, NCLX-mediated Ca^{2+} transport is modulated by multiple monovalent cations (Li^+, Na^+, K^+) and protons, whereas NCX1 activity is affected solely by Na^+. This discrepancy has been briefly addressed in the revised Discussion section.

8. The manuscript proposes that trimerization represents the native oligomeric state of NCLX, mediated by interactions involving TM9/10 and Zn^{2+}-coordinated His581, while the monomeric form is attributed to detergent-induced disruption. It would be important to clarify whether NCLX trimerization has been validated in a native membrane context. For example, have the authors assessed NCLX oligomerization using approaches such as FRET, co-immunoprecipitation, or size-exclusion chromatography of mitochondrial extracts under near-native conditions? In addition, the functional Ca^{2+} uptake assays were conducted using plasma membrane-localized NCLX in GnTI$^-$ cells, which may not reflect its physiological environment or oligomeric state in the mitochondrial inner membrane. If trimerization represents the native and functionally relevant state, does the monomeric structure correspond to a detergent-induced artifact, or could it represent a physiologically relevant form?

To assess the oligomeric state of NCLX in mitochondria, we analyzed the size-exclusion chromatography profile of NCLX from mitochondrial extracts of NCLX-expressing cells (included in the revision as Supplementary Fig. 1c-d). NCLX isolated from mitochondria eluted as two distinct peaks corresponding to trimeric and monomeric fractions. Compared with NCLX purified from total cell extracts, the mitochondrial fraction contained a higher proportion of the trimeric form. These results indicate that the trimeric form of NCLX exists in both mitochondrial and plasma membranes in our expression system. This finding has been included in the revised manuscript.

We speculate that NCLX primarily forms trimers in its native state due to extensive trimerization interactions, although monomeric forms may coexist. Further studies are required to verify the native oligomeric state of NCLX. Notably, NCLX also forms trimers in the study of Fan et al (Fan et al., Nature 2025, PMID: 40931067). The monomeric structure closely resembles a single protomer of the trimer, and the trimerization region does not participate in conformational changes during ion exchange. Thus, both monomeric and trimeric forms are likely functional in the membrane, and the monomeric structure provides a valid framework for understanding the NCLX exchange mechanism.

9. Functional assays show that Li^+, Na^+, and K^+ all inhibit NCLX-mediated Ca^{2+} uptake, supporting its role as a non-selective cation/Ca^{2+} exchanger. However, the stoichiometry of exchange is noted as unresolved. Could the authors comment on whether multiple monovalent cations are transported per Ca^{2+}, and whether this might explain the slower kinetics?

The stoichiometry proposed in our model is inferred from previously characterized Na^+/Ca^{2+} exchangers, including the extensively studied NCX1 and the high-resolution structure of the archaeal homolog NCX_Mj, both of which share conserved structural features with NCLX. A 3:1 Na^+/Ca^{2+} exchange ratio has been well established for NCX through electrophysiological measurements. In NCX, high-affinity Ca^{2+} binding at the S_{Ca} site is progressively weakened by the binding of multiple Na^+ ions, which reduce Ca^{2+} affinity through charge neutralization and electrostatic repulsion, ultimately promoting Ca^{2+} release during the exchange cycle. Given the strong structural conservation of the ion-binding pocket, we propose that NCLX likely adopts a similar 3:1 stoichiometry for monovalent cation/Ca^{2+} exchange. This point has been incorporated into the revised Discussion section.

The relatively slow kinetics observed for NCLX are more likely attributable to slower conformational transitions during the alternating-access cycle, rather than the requirement for multiple ion-binding events.

10. The manuscript references recent studies suggesting that TMEM65, rather than NCLX, plays the determinant role in mitochondrial calcium regulation and export, yet the conclusion continues to emphasize NCLX as a critical regulator without further discussing potential functional redundancy between NCLX and TMEM65 in calcium signaling. Do the authors have evidence for physical or functional interactions between these two proteins? Would TMEM65 knockdown affect the function of NCLX? It is advisable to supplement this content in the discussion section.

Several recent studies have identified TMEM65, another inner mitochondrial membrane protein, as a key contributor to mitochondrial Ca^{2+} regulation and efflux, either directly or through its modulation of NCLX (Garbincius et al., Nature Metabolism, 2025, PMID: 40200126; Vetralla et al., Nature Communications, 2025, PMID: 41408045; Zhang et al., Nature Cell Biology, 2025, 40691517). In one study, TMEM65 has been reported to interact with NCLX and regulate mitochondrial Ca^{2+} efflux in an NCLX-dependent manner (Garbincius et al., Nature Metabolism, 2025, PMID: 40200126). In contrast, two other studies have proposed that TMEM65, rather than NCLX, directly mediates rapid mitochondrial Ca^{2+} extrusion (Vetralla et al., Nature Communications, 2025, PMID: 41408045; Zhang et al., Nature Cell Biology, 2025, 40691517). The relative contributions of TMEM65 and NCLX to mitochondrial Ca^{2+} handling remain debated,

and our study does not resolve this controversy. Our structural and functional data demonstrate that NCLX possesses intrinsic Ca^{2+} exchange activity and likely remains an essential regulator of mitochondrial Ca^{2+} homeostasis. The specific role of TMEM65 in mitochondrial Ca^{2+} extrusion is beyond the scope of this work. We have briefly discussed recent findings on the functional roles of NCLX and TMEM65 in the revised Discussion section.

11. The authors report that NCLX mediates Ca^{2+} exchange with "substantially slower transport kinetics than NCX", yet the structural basis for this kinetic difference remains unclear. They propose that NCLX-specific auxiliary helices (MH3-4, NH1-3), which are partially embedded in the membrane and interact with TM1 and TM6, may restrict the sliding motion of these transmembrane segments. It would be helpful to clarify which specific residues in MH3/MH4 interact with TMs 1/6, how these interactions constrain the "sliding-door" motion during alternating access, and whether deletion these helices accelerate ion exchange. Could molecular dynamics simulations be employed to directly compare the conformational dynamics of these key regions between NCLX and NCX? The physiological significance of the slower transport rate could be discussed in the context of mitochondrial calcium signaling.

The suggestion that the membrane-embedded elements (MH1-4 and NH1-3) contribute to the slow ion exchange of NCLX is speculative, based solely on our model showing their coordinated movement with TMs 1/6, a phenomenon also noted in Fan's study (Fan et al., Nature 2025, PMID: 40931067). Interactions between MH3/MH4 and TMs 1/6 are primarily mediated by MH1/MH2. While other factors may contribute to NCLX's slow kinetics, our structural data cannot fully address this. We did not perform deletions of these well-structured elements, as such deletions would likely compromise protein expression and stability. Furthermore, the slow conformational dynamics of NCLX make them difficult to capture with molecular dynamics simulations. Given the lack of direct experimental evidence, we have removed this speculation from the revised manuscript to avoid potential confusion.

Mitochondrial calcium signaling involves multiple protein factors, and how these collectively regulate mitochondrial Ca^{2+} homeostasis remains debated. Our study focuses on the structural characterization of NCLX and its ability to mediate Ca^{2+} exchange with monovalent counterions. The precise functional role of NCLX within the broader context of mitochondrial calcium signaling remains an open question beyond the scope of this work and deserves future investigation.

Minor concerns:

1. Line numbers should be added to the manuscript.

The line numbers have been added as suggested.

2. The abbreviations of S_{int}, S_{ext}, and S_{mid} are not defined in the manuscript or figure legends. The authors should define these terms at their first occurrence.

The ion-binding sites have been defined in the revised manuscript.

3. In main Figure 2c, the left and right panels clearly show the same view, however, the stick radius and sphere sizes appear inconsistent between the two panels. The authors should correct this issue.

We have corrected it in the revised manuscript.

4. Scale bars need to be added to the micrographs in Fig. S2 and S3.

The scale bars have been added in the revised manuscript.

5. The notation “2X CTF” should be changed to “2 × CTF”; please check the entire manuscript for consistent use of the multiplication sign.

It has been corrected in the revision.

6. The particle-orientation distribution plot in Fig. S3 appears mis-aligned and shows very few particles, please verify that it corresponds to the final reconstruction map.

We have re-aligned the dataset accordingly. Because the reconstruction was performed with C3 symmetry, the angular distribution is displayed in the symmetry-reduced (asymmetric) orientation space. As a result, symmetry-related views are merged into a single representation, which makes the distribution appear to contain fewer particles even though the full dataset was used in the reconstruction.

7. I don't have specific experimental data about how the SEC fraction peak and gel image would change when purifying monomeric NCLX with 1 mM EGTA.

In our study, the monomeric NCLX structure was determined from a sample purified in the presence of 1 mM EGTA. Size-exclusion chromatography of purified NCLX showed identical twin-peak profiles regardless of EGTA presence.

8. Page 5, line7, Fig. 1f should be cited.

We have referenced the figure as suggested in the revision.

9. The figure legend for Fig. S6b-c is mismatched, and the legend for Fig. S6d-e is missing.

We have corrected the mistake in the revision.

10. The role of NH1-3 in conformational changes deserves discussion.

Given the lack of direct experimental evidence, we chose not to speculate on the potential role of auxiliary elements, including NH1-3, in NCLX conformational changes.

11. The legend of Fig. S3d should be corrected from monomeric NCLX to trimeric NCLX.

It has been corrected in the revision.

12. Page 7, line 19. What accounts for the localization of overexpressed NCLX to the plasma membrane in HEK cells instead of its native mitochondrial inner membrane?

Many organelle membrane proteins can partially localize to the plasma membrane in addition to their native organelle membranes when overexpressed in HEK cells. We have observed this phenomenon in multiple studies of organelle membrane proteins. It likely occurs because the cellular trafficking machinery and quality control systems become saturated under overexpression conditions.

13. Page 5, line 26. Does the more open cavity of NCLX permit coordination of divalent cations beyond Ca^{2+}?

Although the cavity of NCLX is slightly more open, the surrounding residues do not provide the proper geometry or chemical environment for Ca^{2+} or other divalent ion binding, except at the S_{Ca} site.

Reviewer #3:

I co-reviewed this manuscript with one of the reviewers who provided the listed reports. This is part of the Nature Communications initiative to facilitate training in peer review and to provide appropriate recognition for Early Career Researchers who co-review manuscripts.

This reviewer co-reviewed the manuscript.

Reviewer #4:

In this study, the authors determined cryo-EM structures of rat NCLX in monomeric and trimeric forms, corresponding to the cytosolic-facing open and occluded conformational states, respectively. By comparison with plasma membrane Na^{+}-Ca^{2+} exchanger NCXs, the authors argue that the central transmembrane module of NCLX adopts an architectural framework conserved among NCX family exchangers; however, the absence of Na^{+}-binding residues renders the exchanger nonselective for monovalent cations during Ca^{2+} exchange. To further substantiate this idea, the authors performed cell-based functional assays, demonstrating that NCLX mediates slower Ca^{2+} uptake than NCX, and exhibits sensitivity not only to Na^{+}, but also to Li^{+}, K^{+}, and H^{+}. Overall, the study is well designed, and the results are interesting and are likely to make a significant contribution to this field. However, several explanations are insufficient, and some of the interpretations appear to be too speculative. Given that structures of rat NCLX in distinct conformational states have been reported recently (Fan et al., Nature, 2025), a more thorough and rigorous comparative structural analysis is necessary. Specific comments are as follows.

We thank the reviewer for the positive evaluation of our work and for the detailed suggestions to improve the manuscript. We have carefully addressed all comments and provided detailed responses below.

Major

1. page 4, 2nd paragraph. "Considering the mitochondrial localization of NCLX, the matrix-facing side corresponds to the extracellular side of NCXs in our comparisons (Fig. S4).". NCX in its forward mode extrudes cytosolic Ca^{2+} in exchange for extracellular Na^{+}, whereas NCLX in its forward mode extrudes matrix Ca^{2+} in exchange for cytosolic Na^{+}. This reviewer is not fully convinced that the present alignment is appropriate. Related to this, the human NCX1 structure (PDB ID: 8SGJ) demonstrated by the present authors captures a Na^{+}-dependent inactivated state, a recognized regulatory feature of NCX1, not of NCLX, that is proposed to lie outside the canonical Na^{+}/Ca^{2+} exchange cycle. In this context, what would the interpretation change if the NCLX structure were aligned with 8SGT, which represents a Ca^{2+}-bound, activated state of NCX1 that is permissive for transport?

We selected the described orientation of NCX and NCLX for structural comparison of the transmembrane (TM) region to ensure proper alignment of the N- and C-terminal halves of NCLX with their counterparts in NCX. Without this orientation, the structural comparison would be inverted, precluding accurate structural alignment. This orientation is specified in the legend of Supplementary Fig. 4 in the revised manuscript.

The TM region of the Ca^{2+}-bound, activated NCX1 structure (PDB ID: 8SGT) is poorly resolved and therefore unsuitable for direct comparison with NCLX. Given the bidirectional nature of ion exchange, the forward and reverse modes of these exchangers are relative definitions.

2. page 4, last paragraph. "This arrangement suggests that these elements form part of a regulatory module with TMs 1 and 6 and are likely to move together as a rigid body during conformational cycling.". Please show data supporting the role of these parts as a "regulatory module".

Our suggestion that the membrane-embedded elements (MH1-4 and NH1-3) may contribute to NCLX ion exchange activity is based solely on their close association with TMs 1 and 6 in our structure, which implies coordinated movement during conformational transitions. A similar observation was reported in Fan *et al* (Fan et al., Nature, 2025, PMID: 40931067) as discussed in our manuscript. However, we currently lack functional data to support a direct role for these elements, and deletion of these well-structured regions would likely compromise protein expression and stability. In the absence of direct experimental evidence, we have revised the manuscript to avoid referring to these elements as a "regulatory module."

3. page 5, 1st paragraph. Please provide supporting data or relevant literature, if available, regarding transition metal-mediated regulation of NCLX.

Regarding the identity of the metal ion, we cannot definitively conclude that it is Zn^{2+}. The density map reveals a strong non-protein density located beneath the histidine cluster, positioned to interact with the side chains of the surrounding histidine residues. The geometry of this density (Fig. 1f) is consistent with histidine-mediated coordination of Zn^{2+}, as observed in some known zinc-binding proteins (Wolfgand Maret, J Inorg Biochem, 2012, PMID: 22196021), and Zn^{2+} is among several divalent metal ions present in the mitochondrial matrix (Liu et al., Int J Physiol Pathophysiol Pharmacol, 2016, PMID: 27186321). Based on these considerations, we tentatively modeled this density as a Zn^{2+} ion. However, we cannot exclude the possibility that it represents another metal ion. This clarification has been incorporated into the revised manuscript.

To evaluate the potential role of the putative Zn^{2+}-binding site, we generated an NCLX mutant lacking residues 581-585. Size-exclusion chromatography revealed a reduced trimer-to-monomer ratio compared with the wild-type protein (Supplementary Fig. 7a-b), indicating that the C-terminal histidine cluster contributes to trimer assembly. Functional analysis of the truncated mutant showed a marked reduction in activity relative to wild-type NCLX (Fig. 5h). Together, these results demonstrate that the C-terminal histidine cluster is critical for both trimer assembly and NCLX function. These findings have been incorporated into the revised manuscript (Fig 5 and Supplementary Fig. 7).

4. The authors argue that the ion density at S_{Ca} site in monomeric NCLX structure corresponds to Na^+, as Ca^{2+} was chelated by EGTA. If this is the case, why is Na^+ not observed around the open cavity in trimeric NCLX structure, which corresponds to S_{int}, S_{mid}, and S_{ext} in NCX_Mj, even though Na^+ is the only monovalent cation present under this condition?

Two key side chains involved in Na^+ binding at the S_{int} and S_{ext} sites of NCX_Mj are absent in NCLX: S236 at S_{int} is replaced by Gly, and S77 at S_{ext} is replaced by Ala. These substitutions likely underlie the loss of Na^+ selectivity in NCLX, allowing other monovalent cations to access the ion-binding pocket as counter ions for Ca^{2+} exchange. We suspect that Na^+ and other monovalent ions enter the open cavity without residing at a defined site, a mode of binding that would not be resolved in our EM structures.

5. Please provide a more detailed discussion comparing the present structure with the reported NCLX structure in the cytosol-facing conformation (Fan et al., Nature, 2025).

As suggested, we have included a structural comparison between our trimeric NCLX and the previously reported cytosolic-facing conformation in the revised manuscript (Supplementary Fig. 5e).

6. Functional analyses. Please clarify the topology of NCLX in the plasma membrane of HEK cells. The authors evaluated the activities of NCX and NCLX in their "reverse mode", i.e., Ca^{2+} influx from extracellular space to cytosol in exchange for cytosolic monovalent cations, possibly Na^{+}. The slowing of Ca^{2+} entry by application of extracellular monovalent cations does not necessarily mean that these cations are directly exchanged for intracellular Ca^{2+}. Please provide direct evidence demonstrating that Ca^{2+} is exchanged for monovalent cations.

The study by Cai and Lytton reported that overexpression of NCLX (referred to as NCKX6 in their study) with a C-terminal FLAG tag in HEK293 cells resulted in robust cell-surface detection by anti-FLAG antibodies without membrane permeabilization (Cai and Lytton, JBC, 2004, PMID: 14625281). This observation indicates that, under overexpression conditions, the C-terminus of NCLX is exposed to the extracellular side. In its native mitochondrial environment, both the C- and N-termini of NCLX face the mitochondrial matrix. Therefore, when localized to the plasma membrane, the matrix-facing side of NCLX is exposed to the extracellular space. The orientation of NCLX and its comparison with NCX have been described in the legend for Supplementary Fig. 4 in the revised manuscript.

All our functional assays were performed alongside NCX1, a well-characterized Na^{+}-dependent Ca^{2+} exchanger, under identical experimental conditions. Although our cell-based competition assays cannot fully exclude the possibility that these monovalent ions act as blockers rather than participating directly in ion exchange, several observations support their role as counter-exchanging ions. First, Ca^{2+} uptake is not completely abolished even at high concentrations of monovalent cations, arguing against simple competitive blockade and instead suggesting that reduced uptake reflects an increased inward gradient of counterions opposing Ca^{2+} influx. Second, in Na^{+}-selective NCX1, only Na^{+} suppresses Ca^{2+} uptake, whereas in NCLX, Li^{+}, Na^{+}, and K^{+} produce comparable inhibitory effects. This makes it unlikely that Na^{+} acts as the exchange ion in NCX1 while these same monovalent cations function as blockers in NCLX. Third, the high structural similarity between NCX1 and NCLX, particularly within the α-repeat regions that mediate ion exchange, supports a conserved alternating-access mechanism. The absence of specific Na^{+}-binding residues in NCLX likely permits broader access of monovalent cations to the ion-binding pocket. Within this framework, monovalent cations that enter the pocket from one side at high concentration are expected to be released to the opposite side during conformational cycling, thereby functioning as counterions in Ca^{2+} transport. We have incorporated this discussion into the revised Discussion section.

7. page 9. "We speculate that membrane-embedded elements closely associated with TMs 1 and 6, but absent in NCX, may restrict their mobility and consequently slow the exchange cycle". It would be interesting to investigate potential functional changes resulting from the removal of mutation of these regions in NCLX.

The suggestion that the membrane-embedded elements (MH1-4 and NH1-3) contribute to the slow ion exchange of NCLX is speculative, based solely on our model showing their

coordinated movement with TMs 1/6, a phenomenon also noted in Fan's study. While other factors may contribute to NCLX's slow kinetics, our structural data cannot fully address this. We did not perform deletions of these well-structured elements, as such deletions would likely compromise protein expression and stability. Given the lack of direct experimental evidence, we have removed this speculation from the revised manuscript to avoid potential confusion.

Minor

page 12. "*in vitro*" rather than "*in vivo*"?

It has been corrected in the revision.

Response to Reviewers

Reviewer #1:

The authors have adequately addressed all major concerns, and I believe the manuscript is now ready for publication.

We are grateful to Reviewer #1 for the positive comments on our manuscript.

Reviewer #2:

The authors have addressed the previous comments by providing additional clarifications, experimental data, and supporting references. These revisions improve the overall clarity and presentation of the manuscript.

We appreciate Reviewer #2's positive and supportive evaluation of our work. The remaining points are addressed below:

Minor concerns:

1. In supplementary Figures 1a and 1c, the horizontal axes share the same scale, but the overall figure dimensions differ substantially. The authors should unify the panel sizes and align the two panels properly.

As suggested, the horizontal axis scales in Supplementary Figures 1a and 1c have been corrected and aligned in the revised manuscript.

2. The legend of Supplementary Figure 6c should be corrected from NCX1 to NCLX1.

This has been corrected in the revision.

3. In the response to major concerns 1, the left and right panels clearly show the same view, however, the displayed size of Zn^{2+} is inconsistent between the two panels. Additionally, the Supplementary Fig. 7a-b cited in the response should be corrected to Supplementary Fig. 7c.

The radius of the Zn^{2+} ion in the figure included in our previous response has been adjusted for consistency, as shown below. We also apologize for incorrectly citing Supplementary Fig. 7a-b in our previous response; the correct reference should be Supplementary Fig. 7c. We have carefully checked the manuscript and ensured that all figure references are correct.

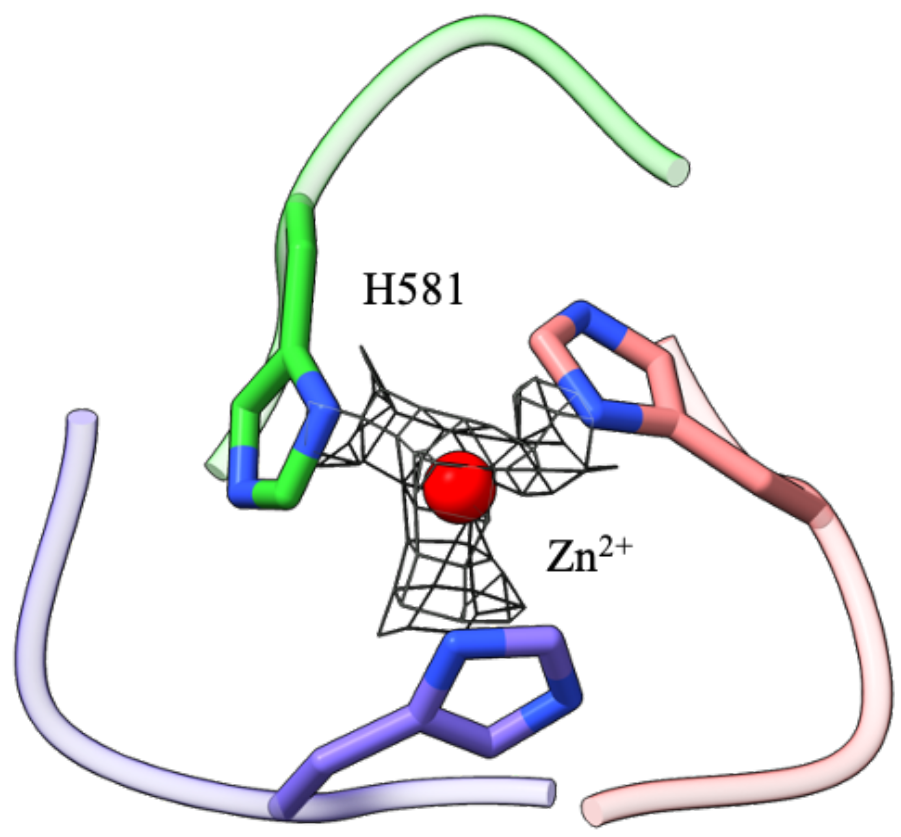

Reconstruction with C3 symmetry

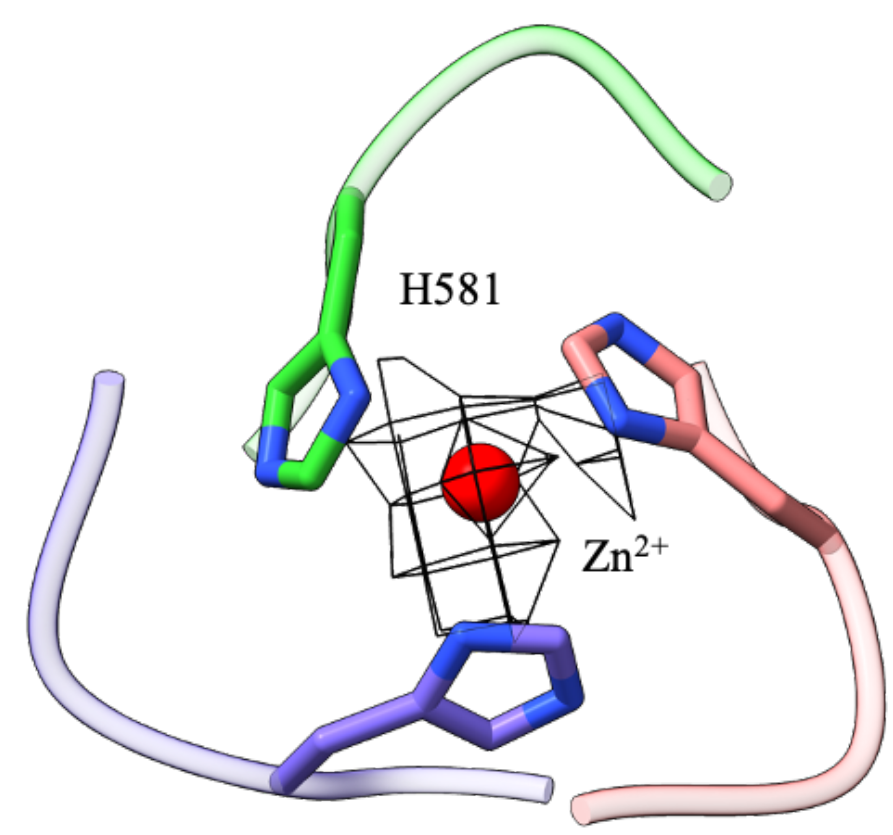

Reconstruction with C1 symmetry

Reviewer #3:

I co-reviewed this manuscript with one of the reviewers who provided the listed reports. This is part of the Nature Communications initiative to facilitate training in peer review and to provide appropriate recognition for Early Career Researchers who co-review manuscripts.

We thank Reviewer #3 for reviewing our manuscript.

Reviewer #4:

I appreciate the authors' efforts in revising the manuscript. While it has been significantly improved in this revision, there are still a few areas where the explanations remain insufficient and require further clarification.

We thank Reviewer #4 for the positive evaluation of our manuscript and for the thoughtful comments. The points raised are addressed below.

Major

1. Supplementary Fig. 4. This reviewer understands that the TM region of the Ca^{2+}-bound, activated NCX1 structure (PDB ID: 8SGT) is poorly resolved and therefore cannot be used for precise analysis. However, as noted previously, the Na^{+}-dependent inactivated state lies outside the canonical Na^{+}/Ca^{2+} exchange cycle. That is, it is not part of the normal transport turnover. It should be referred to in the manuscript.

Indeed, NCLX lacks the cytosolic regulatory domains present in NCX1, including the eXchanger Inhibitory Peptide (XIP) and the two calcium-binding domains (CBDs) responsible for Na^{+}-dependent inactivation. We therefore limited our comparison to the TM regions that mediate

ion exchange activity between NCLX and NCX1. This clarification has been added to the legend of Supplementary Fig. 4.

2. Ca^{2+} uptake assay. It would be helpful to present the uptake kinetics (or rates), in addition to the level of Ca^{2+} accumulation, especially for the mutants.

We appreciate the reviewer's suggestion. However, we are hesitant to quantify the kinetics (or rate) of NCLX-mediated Ca^{2+} uptake for several reasons. Because NCLX exhibits relatively slow transport activity, measurements using Fura-2 fluorescence require several minutes to obtain consistent Ca^{2+} uptake signals. At earlier time points, which would be necessary for accurate rate measurements, the Fura-2 signals are typically noisy, making reliable quantification of uptake rates difficult. In addition, variability between different batches of cells introduces further experimental variation. We therefore chose to quantify Fura-2 signals 5 minutes after Ca^{2+} addition, when uptake had reached a steady state, and the fluorescence signals were substantially above background, resulting in more reproducible measurements.

3. It is practically possible to record NCLX currents using the patch-clamp technique (PMID: 32354321).

We thank the reviewer for pointing out the study reporting NCLX current measurements. However, we believe that the identity of the reported currents from isolated mitoplasts remains uncertain. To our knowledge, NCX1 is the only Na^+/Ca^{2+} exchanger for which measurable currents have been reliably recorded using giant-patch electrophysiology, typically in HEK cells or Xenopus oocytes with very high levels of NCX1 overexpression. Even under those conditions, the measured NCX1 currents are substantially smaller than the currents reported in the above-mentioned mitoplast study, despite the much smaller membrane area and lack of overexpression in the mitoplast preparation. Therefore, considering the relatively slow exchange activity of NCLX, we think it is unlikely that the reported currents originated from endogenous NCLX. In addition, the inhibitor CGP-37157 used in that study has recently been reported to inhibit TMEM65 rather than NCLX in two independent studies (PMID: 40691517 and PMID: 41408045).

Response to Reviewers

All reviewer comments have been fully addressed, and no further revisions are required in response to the reviews.
